# A Mobile Intervention for Self-Efficacious and Goal-Directed Smartphone Use in the General Population: Randomized Controlled Trial

**DOI:** 10.2196/26397

**Published:** 2021-11-23

**Authors:** Jan Keller, Christina Roitzheim, Theda Radtke, Konstantin Schenkel, Ralf Schwarzer

**Affiliations:** 1 Department of Education and Psychology Freie Universität Berlin Berlin Germany; 2 Department of Psychology Humboldt Universität zu Berlin Berlin Germany; 3 Institute of Psychology University of Wuppertal Wuppertal Germany; 4 Department of Psychology University of Zurich Zurich Switzerland; 5 SWPS University of Social Sciences and Humanities Wroclaw Poland

**Keywords:** problematic smartphone use, smartphone unlocks, smartphone time, behavior change, self-efficacy, action planning, digital detox, time-out, randomized controlled trial

## Abstract

**Background:**

People spend large parts of their everyday life using their smartphones. Despite various advantages of the smartphone for daily life, problematic forms of smartphone use exist that are related to negative psychological and physiological consequences. To reduce problematic smartphone use, existing interventions are oftentimes app-based and include components that help users to monitor and restrict their smartphone use by setting timers and blockers. These kinds of digital detox interventions, however, fail to exploit psychological resources, such as through promoting self-efficacious and goal-directed smartphone use.

**Objective:**

The aim of this study is to evaluate the theory-based smartphone app “Not Less But Better” that was developed to make people aware of psychological processes while using the smartphone and to support them in using their smartphone in accordance with their goals and values.

**Methods:**

In a randomized controlled trial, effects of a 20-day intervention app consisting of five 4-day training modules to foster a goal-directed smartphone use were evaluated. In the active control condition (treatment as usual), participants received a digital detox treatment and planned daily time-outs of at least 1 hour per day. Up to a 3-week follow-up, self-reported problematic smartphone use, objectively measured daily smartphone unlocks, time of smartphone use, self-efficacy, and planning towards goal-directed smartphone use were assessed repeatedly. Linear 2-level models tested intervention effects. Mediation models served to analyze self-efficacy and planning as potential mechanisms of the intervention.

**Results:**

Out of 232 enrolled participants, 110 (47.4%; 55 participants in each condition) provided data at postintervention and 88 (37.9%; 44 participants in each condition) at 3-week follow-up. Both conditions manifested substantial reductions in problematic smartphone use and in the amount of time spent with the smartphone. The number of daily unlocks did not change over time. Further, modelling changes in self-efficacy as a mediator between the intervention and problematic smartphone use at follow-up fit well to the data and showed an indirect effect (*b*=–0.09; 95% bias-corrected bootstrap CI –0.26 to –0.01), indicating that self-efficacy was an important intervention mechanism. Another mediation model revealed an indirect effect from changes in planning via smartphone unlocks at postintervention on problematic smartphone use at follow-up (*b*=–0.029, 95% bias-corrected bootstrap CI –0.078 to –0.003).

**Conclusions:**

An innovative, theory-based intervention app on goal-directed smartphone use has been found useful in lowering problematic smartphone use and time spent with the smartphone. However, observed reductions in both outcomes were not superior to the active control condition (ie, digital detox treatment). Nonetheless, the present findings highlight the importance in promoting self-efficacy and planning goal-directed smartphone use to achieve improvements in problematic smartphone use. This scalable intervention app appears suitable for practical use and as an alternative to common digital detox apps. Future studies should address issues of high attrition by adding just-in-time procedures matched to smartphone users’ needs.

**Trial Registration:**

German Clinical Trials Register DRKS00017606; https://tinyurl.com/27c9kmwy

## Introduction

After the first iPhone was released in 2007, smartphones have become an integral part of people’s everyday life. Worldwide, in 2021, 3.8 billion persons are using a smartphone [1] and spend large parts of their leisure time with their smartphone (eg, reading news, social media, chatting with friends [2]). Smartphones are used for a variety of daily tasks, thereby simplifying life in many ways. However, there is an increasing scientific and public debate on problematic forms of smartphone use [3]. Empirical findings show links of problematic smartphone use with psychopathology, such as depression, anxiety, stress, and sleep disturbances, as well as negative physical consequences, such as forward neck posture and hand dysfunction [4-7]. Problematic smartphone use can be defined as the “inability to regulate one’s use of the mobile phone, which eventually involves negative consequences in daily life” [8]. For instance, this “inability” or lack of control about one’s smartphone use can manifest through habitual smartphone checking [4], which occurs on average 88 times per day [9]. Due to high and increasing prevalence rates, problematic smartphone use is considered to be an emerging public health problem [10].

To reduce problematic smartphone use, behavioral approaches focus on either complete abstention or moderating smartphone use by cutting it down—so called digital detox interventions [10]. Several technology-based solutions are available including smartphone apps which help users to monitor and restrict their use by setting timers and blockers. However, most apps lack a psychological underpinning and have not been evaluated by trial designs [10]. Another issue is that monitoring and restrictions alone might not be sufficient as indicated by several studies that examined digital detox interventions [11,12]. Empirical evidence shows that daily smartphone time-outs can indeed lead to decreases of smartphone use [13]; however, there are mixed findings regarding the effects on psychological outcomes [12]. Whereas some studies reveal that digital detox interventions are not related to psychological factors such as well-being or cognitive performance [12], some studies show even negative effects (eg, decreased life satisfaction, lowered affect, or an increase in loneliness) [14-16]. Digital detox interventions might not address useful psychological resources, such as those that can promote a self-efficacious and goal-directed smartphone use, which would be crucial to achieving sustainable behavioral changes [17].

Psychological resources were addressed in an existing group-based intervention app, which included self-monitoring, goal setting, social learning, and competition as active ingredients [18]. Findings from this intervention study showed that daily smartphone use in the intervention condition decreased from 234 to 177 minutes and smartphone-related self-efficacy beliefs were significantly promoted by the intervention [18]. Although these findings seem promising, more research is needed to investigate the mechanisms of these kinds of resource-oriented interventions. Given the previous literature, it remains unclear whether psychological resources (eg, self-efficacy) increased by such interventions would lead to improvements in target outcomes such as problematic smartphone use [4].

The intervention app “Not Less But Better” was developed which focuses on the promotion of psychological resources for goal-directed smartphone use within a 20-day program and is tailored to individuals’ goals and values. The intervention offers techniques grounded in cognitive behavior therapy, acceptance and commitment therapy [19], and health behavior change theories like the health action process approach (HAPA) [20,21]. Acceptance and commitment therapy involves allowing unwanted thoughts, feelings, and urges to come and go without struggling with them, and setting value-based goals and achieving them. HAPA reflects a sequence of motivational and volitional constructs, in particular self-efficacy and planning, that are likely to support people in translating their behavioral goals into action. Based on these theoretical frameworks, several behavior change techniques (BCTs) [22]; that is, the smallest units of interventions that can induce behavior change, are applied by the intervention app. These BCTs include promoting self-efficacy beliefs to use the smartphone in accordance with personal goals (eg, focus on past success, BCT 15.3; or vicarious reinforcement, BCT 16.3) and planning when, where, and how to use the smartphone (ie, action planning, BCT 1.4) [22]. The active control condition comprises a 20-day digital detox intervention (ie, treatment as usual) with daily time-out restrictions of at least 1 hour per day (eg, not using the smartphone from 6 pm to 7 pm). This active control condition is in line with common procedures used in digital detox interventions [12,13].

The first aim of our study was to evaluate the effectiveness of the intervention condition in decreasing problematic smartphone use, daily smartphone unlocks (as an indicator for smartphone checking), and time of daily smartphone use. Extending previous studies on digital detox interventions [12], the second aim explored the psychological mechanisms of the intervention through comparison with the active control condition.

In relation to the primary outcome, we hypothesized that problematic smartphone use would show higher decreases in the intervention condition than in the active control condition.

In relation to other outcomes, changes over time of 2 behavioral indicators of goal-directed smartphone regulation were tested: (1) the frequency of daily smartphone unlocks and (2) the time of daily smartphone use. Persons in the intervention condition received psychological strategies to use their smartphone when in accordance with their goals, whereas persons in the active control condition were restricted to not use their smartphone within the self-set time-out interval. We hypothesized that daily smartphone unlocks and time of daily smartphone use would show reductions in both conditions and that no between-group differences would be present (equivalence hypothesis).

Regarding intervention mechanisms, possible pathways of how the intervention condition is related to reductions in problematic smartphone use via self-efficacy and planning of goal-directed smartphone use and reduced smartphone unlocks (as an indicator for smartphone checking behavior) were explored.

## Methods

### Study and Approval

This study reports primary findings from an app-based, 2-condition, randomized controlled trial (RCT) on healthy smartphone use among adults from the general population. The preregistration for the RCT can be accessed at the German Clinical Trials Register (DRKS00017606; date of registration: August 9, 2019; first participant enrolled: October 9, 2019; targeted sample size: 200). To provide a deeper focus on smartphone-related outcomes and intervention mechanisms, this paper reports findings on the primary outcome (problematic smartphone use), whereas findings on the secondary outcome of the RCT, psychological well-being, are not reported. The Ethics Committee of the Humboldt Universität zu Berlin granted ethics approval for this study (registration #2019-14R1).

### Recruitment and Design

Eligible participants were at least 18 years old, owned and used a smartphone with an iOS operating system (minimum Apple iPhone 5, iOS system 10+), and had sufficient visual ability and skills to understand and complete the English language study materials. Participants were recruited by using reactive strategies such as flyers, online postings, and email lists. As an incentive for study participation, participants took part in a lottery of 4 online shopping vouchers worth €25 (US $29) each and received course credits if needed. Data collection ranged between October 2019 and December 2019.

After downloading the study app and providing informed consent, participants responded to the baseline questionnaire. Subsequently, they were randomly assigned to either the intervention (intervention=1) or active control condition (control=0) using a simple (“flipping a coin”) randomization procedure via a web-based tool. No blinding procedures were used. Based on randomization and throughout the following 20 days (D; D1-D20), participants received daily app-based sessions on goal-directed smartphone use in 5 modules each spanning 4 days (intervention condition) or on defining daily time-outs (active control condition).

**Figure 1:**
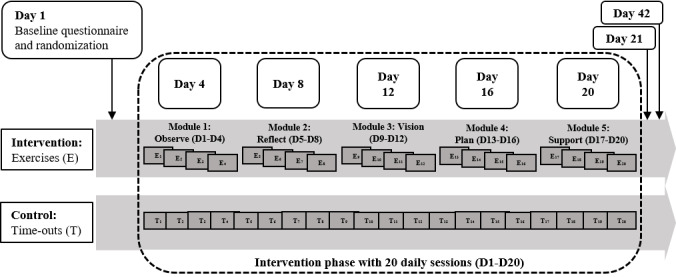
Study design with measurement points and 20 daily sessions of the intervention and active control conditions.

Throughout the 20-day intervention period, participants completed brief questionnaires on D4, D8, D12, D16, and D20, corresponding to the completion of the 4-day modules from the intervention condition. Moreover, participants responded to longer questionnaires at postintervention (D21) and at a 3-week follow-up (D42; Figure 1). Multimedia Appendix 1 (Figure S1) provides a Consolidated Standards of Reporting Trials (CONSORT) flow diagram.

### Intervention

To foster usability and acceptability, the development of the content in the intervention condition followed a user-centered design and person-based approach [23,24]. Initially, a large pool of brief daily exercises was developed based on elements used in cognitive behavior therapy, acceptance and commitment therapy^16^, and behavior change interventions [22]. The pool of exercises was tested with a total of 44 volunteering smartphone users. Moreover, guidance from 7 experts was received, which resulted in a final set of 20 exercises. The material of 5 out of 20 daily exercises (duration: 2 to 10 minutes per day) from the intervention app can be found in Multimedia Appendix 2 and 3.

The five 4-day modules were the following: Observe (D1-D4), Reflect (D5-D8), Vision (D9-D12), Plan (D13-D16), and Support (D17-D20). In the Observe module, participants observed their physical reactions (eg, posture, impulses of checking behavior) to the smartphone in exercises on impulse control and mindfulness. The Reflect module included educational elements regarding a better understanding of habitual and problematic forms of participants’ smartphone use [10]. In the Vision module, smartphone-related exercises on mindfulness, value-related committed action [19], and goal-setting were conducted [20]. The Plan module focused on strategies toward goal-directed smartphone use, such as developing if-then plans on when, where, and how to use the smartphone (action planning) or executing alternative behavioral responses in critical situations (coping planning) [25]. In the Support module, practical tips to support sustainable behavior change (eg, redesign of the home screen) were provided.

Similar to earlier digital detox intervention studies [12], participants in the active control condition received a daily time-out treatment. Across the intervention phase (D1-D20), they were asked to plan a smartphone time-out of at least 1 hour within the next 24 hours. Participants could freely choose whether and how long they executed their planned smartphone time-out; that is, nonaccess to the smartphone was not technically enforced.

### Measures

#### Problematic Smartphone Use

As the primary outcome of the RCT, self-reported problematic smartphone use was measured with 8 items from the Mobile Phone Problem Use Scale [26]) at baseline, postintervention (D21), and follow-up (D42). Items such as “In the past 7 days, I felt anxious if I have not checked for messages or switched on my smartphone for some time” or “In the past 7 days, I have been told that I spend too much time on my smartphone” were answered on a 6-point scale (1=”not at all true” to 6=”exactly true”). Internal consistency across measurement points and conditions ranged between Cronbach’s α=.68 and α=.88.

#### Daily Smartphone Unlocks and Daily Minutes of Smartphone Use

The frequencies of daily smartphone unlocks and daily minutes of smartphone use from the previous 7 days were assessed by asking participants to transfer objectively measured values from the iOS app “Screen Time” (on iOS phones by default) into the study app. At baseline, postintervention, and follow-up, participants responded to the items “What is your average daily number of unlocks of the last 7 days?” (daily smartphone unlocks) and “What is your average screen time per day of the last 7 days?” (daily minutes of smartphone use). Univariate outliers (*z*>3.29) of smartphone unlocks and minutes per day were winsorized to 1 unit higher than the next highest value in the distribution [27].

#### Planning and Self-Efficacy Toward Goal-Directed Smartphone Use

Participants responded to items on planning and self-efficacy toward goal-directed smartphone use on a 6-point scale (1=”not at all true” to 6=”exactly true”) at baseline and throughout the intervention period on D4, D8, D12, D16, and D20. With the instruction “Please refer to today and the past 3 days,” responses referred to days when respective modules were conducted in the intervention condition. Planning and self-efficacy items were adapted from scales that were previously validated in various health behavior settings (eg, dietary behavior, physical activity) [28].

Self-reported planning of goal-directed smartphone use was measured with 5 items using the stem “I have made a detailed plan regarding…” followed by statements such as “when to use my smartphone consciously (eg, “on the way to work)”.

Self-reported self-efficacy toward goal-directed smartphone use was assessed using 3 items with the stem “I am confident that I can…” followed by statements such as “I use my smartphone consciously even if I first have to find a way to integrate this into my daily routine.” Across measurement points and conditions, the internal consistency of the planning scales ranged between Cronbach’s α=.87 and α=.97, whereas self-efficacy scales showed a range of Cronbach’s alpha between α=.84 and α=.95.

#### Perceived Impact of the 20-Day Program and Covariates

To evaluate intervention fidelity, perceived impact of the 20-day program was measured at postintervention using an adapted version (eg, “The app increased my intentions/motivation to address my smartphone use”) of a validated scale developed to assess the quality of mobile health apps [29]. Internal consistency of the 6-item perceived impact scale was α=.87 in the intervention and α=.91 in the active control condition.

The list of covariates comprised participants’ sex, baseline age, smartphone-related action control (scale adapted; eg, “I have tried hard to use my smartphone consciously” [30]), and problematic smartphone use. As a result of attrition analyses (see the Results section), the latter 3 measures were added to the list of covariates to control for selective attrition [31].

#### Statistical Analysis

Power analysis with G*Power version 3.1 revealed that 34 participants per group would be needed to detect a significant within-between interaction (*f*=0.25) in problematic smartphone use (*α*=.05, power=0.80, and *r*=0.40 among repeated measures [26]) across 3 assessments.

Data were analyzed based on the intention-to-treat approach. For applied analyses, Mplus 8 and its full information maximum likelihood procedure were used to account for missing data [32].

Linear 2-level models with 3 time points (D1, D21, and D42; within level) nested in participants (between level) were computed (for a conceptual model see Figure S2, Multimedia Appendix 1). For problematic smartphone use as the outcome (model A), time (linear day trend, centred at 0) x experimental condition (0=active control condition; 1=intervention condition) interactions were estimated. For daily smartphone unlocks and daily time of smartphone use as outcomes, the equivalence hypothesis was tested by comparing a null model (ie, with the day trend as predictor; model B and model C) with a nested alternative model (ie, with addition of the condition and the linear day x condition interaction as predictors). Using log-likelihood parameters of both models, a chi-square difference test was run [33]. A nonsignificant chi-square value would indicate that the null model was better fit to the data, confirming the equivalence hypothesis. Moreover, grand-mean centered covariates were added as between-level predictors. Unless models did not converge, the linear day trend and the linear day trend x experimental condition interaction were modeled as random effects predictors [34].

Regarding mediation models, a simple mediation model (model A) was specified, in which self-efficacy toward goal-directed smartphone use was a putative postintervention (D20) mediator between experimental conditions and follow-up problematic smartphone use (D42). By using the measure of self-efficacy on D20, the effects of all sessions of the active control and intervention conditions on changes in self-efficacy were tested. In a second mediation model (model B), planning toward goal-directed smartphone use and the frequency of smartphone unlocks were tested as putative sequential mediators between experimental conditions and follow-up problematic smartphone use (D42). To assure temporal order of the sequential mediators planning and unlocks (at postintervention; D21), planning reports on D16 and D20 were used to compute a mean score, reflecting planning levels that referred to the days of the fourth and fifth module. In each mediation model, we controlled for the set of covariates and for baseline levels of the mediators and outcome. Model fit was evaluated using the *χ^2^* test statistic, the comparative fit index (CFI), root mean square error of approximation (RMSEA), and the root mean square residual (SRMR), with nonsignificant *P* values of the *χ^2^* test, CFI levels >0.95, and RMSEA and SRMR levels <0.05 indicating good fit [35]. The 95% bias-corrected bootstrap CIs (CI_bc_) of direct and indirect effects were generated by bootstrapping with 5000 resamples.

## Results

### Sample Characteristics, Randomization, and Attrition Check

Randomization to the 2 experimental arms was based on 232 enrolled individuals (205 women, 23 men, 1 diverse, 3 missing values) with a mean age of 29.62 years (SD 8.09, range 18-60 years). Further baseline sample characteristics are displayed in Multimedia Appendix 1 (Table S1).

After providing informed consent and responding to the baseline questionnaire, 114 participants were assigned to receive the intervention on goal-directed smartphone use, and 118 participants were assigned to receive the time-out treatment in the active control condition on D1. A randomization check (*χ2* and *t* tests, followed by logistic regressions) using the experimental condition variable as the outcome revealed no unique between-condition differences in baseline variables, pointing to a successful randomization.

A subsample of 110 (47% out of 232; n=55 in each condition) participants provided data at postintervention, and 88 (38% out of 232; n=44 in each condition) participants did so at the follow-up. Attrition rates within the range of this study are normal for online interventions because researchers do not have much control over the attrition of anonymous participants [36]. Participants from the longitudinal sample (n=88) showed a high response rate to questionnaires between D1 and D42 with a mean response rate of 93% (7.47 out of 8 assessments; SD 0.96, range 4-8).

To examine attrition bias, *χ2* tests, *t* tests, and logistic regressions were performed across baseline variables as well as baseline variable x experimental condition interactions, with a dummy-coded attrition variable (0=dropped out; 1=remained in the study) as the outcome. A significant, unique difference emerged for age (dropped out: mean 31.11 years, SD 8; remaining in the study: mean 27.28 years, SD 7.7), baseline problematic smartphone use (dropped out: mean 3.74, SD 0.77; remaining in the study: mean 3.44, SD 0.75), and baseline action control (dropped out: mean 2.21, SD 1.01; remaining in the study: mean 2.55, SD 1.08). This indicates that participants in the longitudinal sample were younger and demonstrated lower problematic smartphone use and higher action control at baseline when compared to those who dropped out. Subsequent analyses therefore controlled for attrition variables of age, problematic smartphone use, and action control [31].

### User Engagement and Perceived Impact

Regarding user engagement of the total sample (N=232), participants executed on average 11.34 (SD 7.87) daily time-out sessions in the active control condition compared to 12.84 (SD 7.41) daily exercise sessions in the intervention condition (Table S2, Multimedia Appendix 1). No between-conditions differences were found (*F*_1,230_=2.24; *P*=.14; *η*^2^=0.01). A high user engagement across those who were retained for analyses on D21 (n=110) was found, with an average completion rate of 93% of the sessions (18.64 out of 20; SD 3.42) in the active control condition and 97% of the exercises (19.40 out of 20; SD 1.55) in the intervention condition. Between-condition differences in postintervention levels of perceived impact revealed that participants reported a higher impact of exercises in the intervention condition (mean 4.88, SD 0.80) as opposed to the active control condition (mean 3.76, SD 1.21; *F*_1,101_=30.53; *P*<.001; *η*^2^=0.23).

### Changes in Study Outcomes Over Time

**Figure 2:**
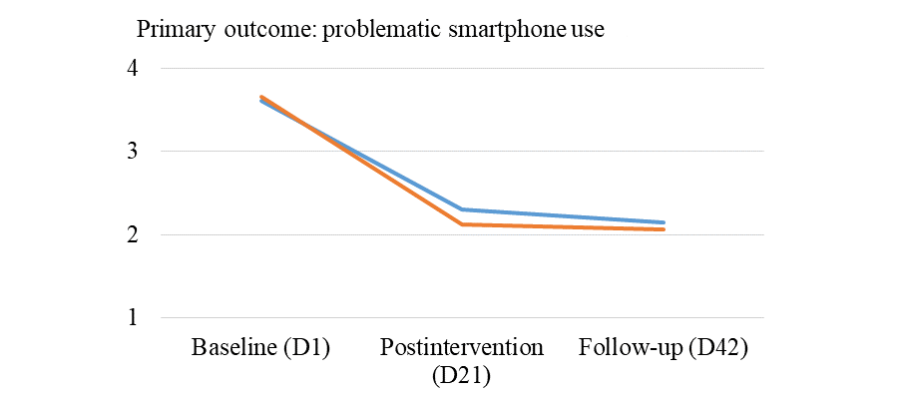
Mean levels of problematic smartphone use in both experimental conditions up to the follow-up. D: study day.

In a first step, mean levels of problematic smartphone use (Figure 2) and additional study variables (Figure S3, Multimedia Appendix 1) over time and across both conditions were visualized.

In the 2-level model with problematic smartphone use as the outcome, a negative linear day prediction was found, indicating that problematic smartphone use decreased over time in the active control condition (Table 1; model A: *b*=–0.04; 95% CI –0.04 to –0.03; intraclass correlation [ICC]=0.21). Not in line with our hypothesis on the primary outcome, the nonsignificant linear day x condition prediction indicated that problematic smartphone use showed a similar decrease over time in the intervention (vs control) condition. Descriptive analyses (Table S2; Multimedia Appendix 1) showed that problematic smartphone use changed from mean 3.60 to mean 2.30 in the active control condition (ie, a decrease of 36%) and from mean 3.65 to mean 2.12 in the intervention condition (ie, a decrease of 42%) throughout the intervention period.

Moreover, daily smartphone unlocks did not change over time (model B: *b*=–0.13; 95% CI –0.29 to 0.03; ICC=0.65), whereas daily time of smartphone use decreased over time (model C: *b*=–0.77; 95% CI –1.12 to –0.43; ICC=0.67). In the testing of the equivalence hypothesis, alternative models with the intervention condition variable as an additional moderator did not yield a better fit to the data when compared to null models (model B: Δ*χ2*_3.15_, Δdf=4, *P*=.53; model C: Δ*χ2*_2.14_, Δdf=4, *P*=.71) [33]. In the alternative models, linear day trend x condition interactions were nonsignificant predictors of daily smartphone unlocks (*b*=–0.15; 95% CI –0.49 to 0.20) and daily time of smartphone use (*b*=0.36; 95% CI –0.32 to 1.05). This indicates that the intervention condition showed similar patterns of change over time in daily smartphone unlocks and daily time of smartphone use when compared to the active control condition.

Regarding significant predictions of covariates, daily smartphone unlocks were more likely when participants reported higher levels of problematic smartphone use at baseline or when they were younger or male; note that only a small group of men (n=23) participated in the RCT. Moreover, a longer duration of smartphone use was more likely when participants reported higher levels of action control and problematic smartphone use at baseline.

**Table 1:** Estimates for 2-level models predicting changes in study outcomes up to a 3-week follow-up (N=228).

Predictors				Model A^a^: problematic smartphone use					Model B^b,c^: smartphone unlocks per day					Model C^a,c^: smartphone use (minutes per day)		
				*b*		95% CI_BC_^d^		*b*			95% CI_BC_		*b*			95% CI_BC_
**Fixed effects**																
	Intercept at baseline			*3.54* ^e^		3.39 to 3.70		*77.94*			72.70 to 83.19		*219.94*			208.67 to 231.22
	Intervention (vs active control)			0.01		–0.19 to 0.21		N/A^f^			N/A		N/A			N/A
	Linear day trend			–*0.04*		–0.04 to –0.03		–0.13			–0.29 to 0.03		–*0.77*			–1.12 to –0.43
	Linear day trend x intervention			–0.01		–0.01 to 0.01		N/A			N/A		N/A			N/A
	Age			–0.01		–0.01 to 0.01		–*1.04*			–1.86 to –0.23		–1.16			–2.51 to 0.19
	Sex (0=female; 1=male)			–0.09		–0.36 to 0.18		*27.66*			*6.43 to 48.89*		7.81			–23.15 to 38.77
	Acton control at baseline			–0.05		–0.15 to 0.04		–1.01			–5.18 to 3.16		*11.31*			2.17 to 20.46
	PSU^g^ at baseline			N/A		N/A		*10.99*			4.98 to 17.00		*42.60*			30.76 to 54.45
**Random effect variances**																
	**Level 2 (between person)**															
		Intercept	0.25		0.10 to 0.39		1014.34			518.36 to 1510.32		3938.15			2432.40 to 5443.91	
		Linear day trend	0.01		–0.01 to 0.01		0.06			–0.46 to 0.58		0.15			–1.26 to 1.56	
	**Level 1 (within person)**															
		Residual variance	0.41		0.33 to 0.48		564.95			331.07 to 798.83		2205.84			1372.03 to 3039.64	

^a^Based on 684 observations.

^b^Based on 683 observations.

^c^Based on the equivalence hypothesis, this model was estimated without a linear day x intervention moderation.

^d^CI_BC_: bias-corrected bootstrap CI.

^e^Italics indicate significant fixed effects predictions.

^f^N/A: not applicable.

^g^PSU: problematic smartphone use.

**Figure 3:**
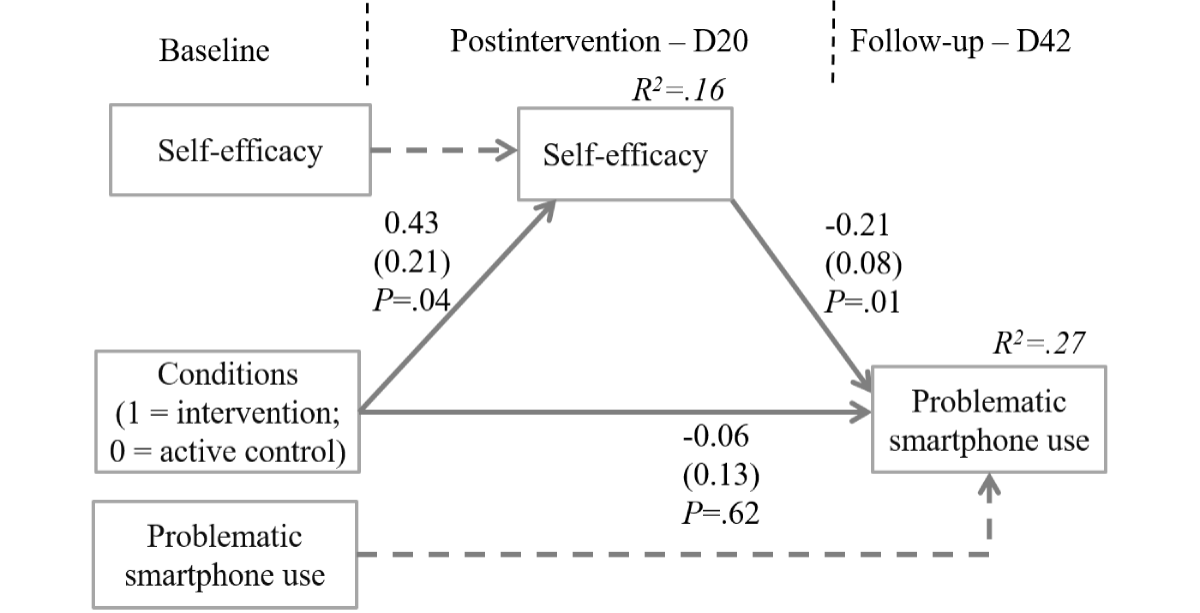
Self-efficacy as a mediator between the intervention condition and problematic smartphone use (unstandardized coefficients).

A simple mediation analysis involving experimental conditions, self-efficacy as the mediator at postintervention (D20), and problematic smartphone use as the outcome at follow-up (D42) was run (Figure 3). This mediation model including data from 231 participants fit well with the data (*χ^2^_8_*=7.06, *P*=.53; CFI=1.00; RMSEA <0.01; SRMR=0.03). The intervention (vs active control) condition was positively related to changes in self-efficacy at postintervention (*b*=0.43; SE=0.21; *P*=.04; 95% CI_bc_ 0.01-0.85) which, in turn, were negatively linked to changes in follow-up problematic smartphone use (*b*=–0.21; SE=0.08; *P*=.01; 95% CI_bc_ –0.36 to –0.05). The mediation yielded a significant indirect effect of *b*=–0.09; 95% CI_bc_ –0.26 to –0.01). Thus, self-efficacy changes at the end of the intervention translated into substantial reductions in problematic smartphone use at 3 weeks following the intervention. Of the variance of self-efficacy and problematic smartphone use, 16% and 27% were accounted for by the joint set of predictors, respectively.

**Figure 4:**
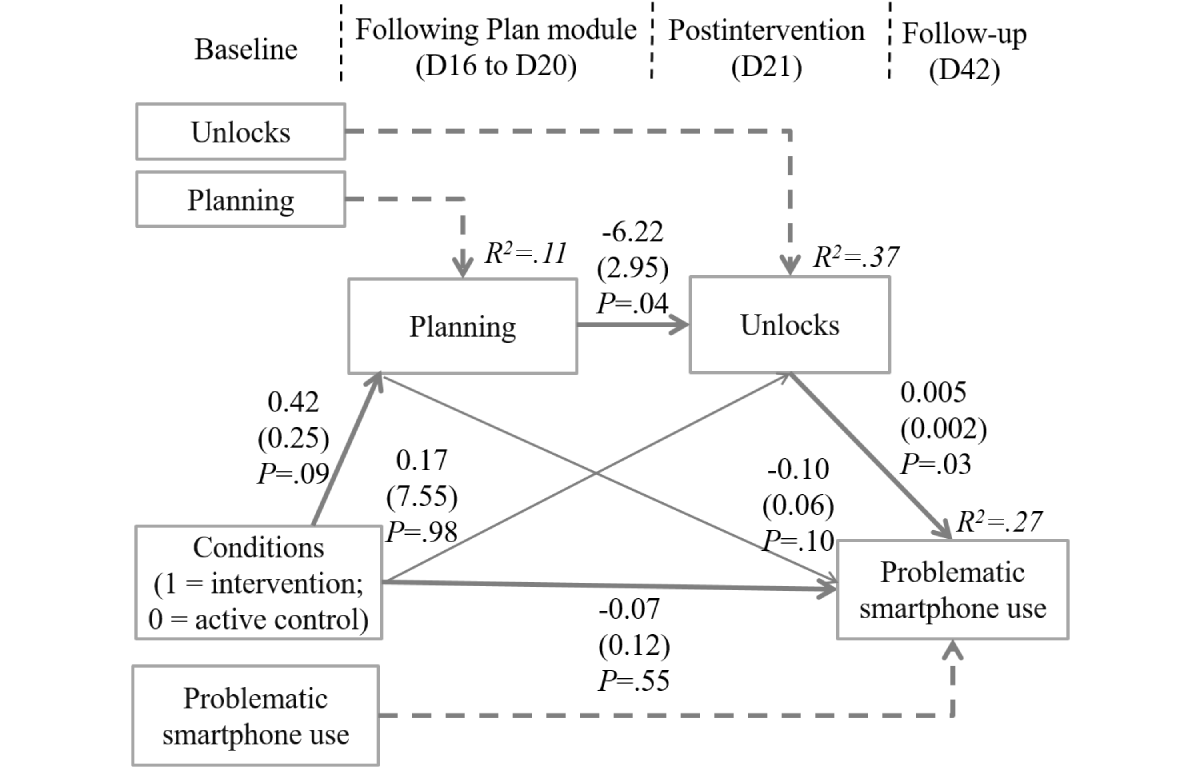
Planning and smartphone unlocks as sequential mediators between the intervention condition and problematic smartphone use (unstandardized coefficients).

A second mediation model was specified with planning after the Plan module (D16 to D20) and smartphone unlocks at postintervention (D21) as sequential mediators (Figure 4) that fit well with the data (*χ^2^*_15_=19.00; *P*=.21; CFI=0.98; RMSEA=0.03; SRMR=0.05). No between-condition differences for changes in planning were found (*b*=0.42; SE=0.25; *P*=.09). Changes in planning across both conditions were related to unlocks at postintervention (*b*=–6.22; SE=2.95; *P*=.04); that is, with each unit of higher planning toward goal-directed smartphone use, the frequency of smartphone unlocks decreased by approximately 6 units per day. Moreover, changes in daily smartphone unlocks across both conditions were significantly related to problematic smartphone use at follow-up (*b*=0.005; SE=0.002; *P*=.03). For the sequential mediation between intervention condition and problematic smartphone use via planning and daily smartphone unlocks, the 95% CI included 0.000 (95% CI_bc_ –0.050 to 0.000), whereas the 90% CI did not include 0 (*b*=–0.012; 90% CI_bc_ –0.043 to –0.002). When testing the simple mediation between planning and problematic smartphone use via daily smartphone unlocks, we found a significant indirect effect (*b*=–0.029; 95%CI_bc_ –0.078 to –0.003). This indicates that higher levels of planning in both conditions at the end of the intervention phase were associated with lower daily smartphone unlocks which, in turn, were connected with problematic smartphone use. The joint set of predictors explained 11%, 37%, and 27% of the variance in planning, daily smartphone unlocks, and problematic smartphone use, respectively.

## Discussion

### Principal Results

An innovative, theory-guided intervention app was field-tested in an RCT by examining a sample of 232 participants up to a 3-week follow-up. The purpose of the app was to make people aware of their (problematic) smartphone use and to support them in establishing self-efficacious and goal-directed smartphone use in their daily life.

The results indicated that the intervention app was useful in lowering problematic smartphone use (primary outcome) as well as time spent with the smartphone. However, observed reductions in both outcomes were not superior to the active control condition (ie, digital detox treatment).

Findings on reductions of problematic smartphone use in both conditions are in line with previous evidence that interventions on psychological resources [18], along with digital detox interventions [37], can be beneficial for health-related outcomes. The findings revealed selective attrition related to higher levels of problematic smartphone use, and thus future interventions targeting problematic forms of smartphone use should add elements so that persons with severe smartphone-related issues receive support matched to their needs. Other strategies to prevent selective attrition in such online-based study designs include scheduling reminders and following up participants with lower study engagement using just-in-time messages or phone calls [38]. Regarding the time of smartphone use, our findings are consistent with evidence from interventions on goal-directed smartphone use [18] and smartphone time-outs [13], thereby testing both forms of interventions concurrently. Although planning a time-out of at least 1 hour, participants in the active control condition reduced their smartphone time by only 43 minutes per day (from 218 to 175 minutes per day; Table S2, Multimedia Appendix 1). Possibly, participants did not fully adhere to their time-out interval or compensated the abstinence from the smartphone by, for instance, catching up with new messages after their time-out [39]. Participants in the intervention condition, who learned and exercised on goal-directed smartphone use, reduced their smartphone time by 32 minutes per day (from 228 to 196 minutes per day; Table S2, Multimedia Appendix 1), an interesting finding given that less smartphone use was not primarily focused on by the intervention app.

To explain the observed changes in problematic smartphone use, intervention mechanisms were systematically examined. According to theories such as the HAPA model [21], self-efficacy toward goal-directed smartphone use should play a role in the process of behavior change. Similar to previous evidence on resource-oriented interventions [18], the present intervention was successfully fostering self-efficacy beliefs which, in turn, are a relevant resource for reductions in problematic smartphone use. The finding that fostering self-efficacious smartphone use is important to improving problematic smartphone use highlights how resource-oriented interventions can even outperform common digital detox interventions. Future interventions targeting problematic forms of smartphone use should follow up on present evidence by enabling persons to control their smartphone use by themselves.

Moreover, precise planning on when, where, and how to use one’s smartphone should also make a difference in behavior change because habitual checking behavior might be reduced [4]. Participants made plans on their smartphone use during the fourth training module (after D13), which was related to postintervention smartphone unlocks; that is, 1 unit of higher planning was linked to a lower daily unlock frequency of 6 units. Planning of smartphone use might result in the planning of smartphone sessions, in which persons take their time to use their phone for current smartphone-related tasks or leisure time activities. This, in turn, might reduce urges towards smartphone-checking behavior and thereby reduce the amount of unlocking of one’s phone. Moreover, the results indicate that daily unlocks at postintervention were linked with problematic smartphone use at follow-up. Next to addressing the time of smartphone use, future research should additionally focus on daily smartphone unlocks—as an indicator of checking behavior—and link smartphone unlocks to clinical-, health-, and work-related outcomes [4,40].

Overall, in terms of practical implications, the findings suggest following up with the currently existing version of the app as a means to change problematic smartphone use by scaling the app.

### Strengths, Limitations, and Future Research

This study has several strengths. A comprehensive 20-day intervention app was evaluated by contrasting it with a treatment-as-usual control condition with various smartphone-related outcomes and testing intervention mechanisms being examined. However, some limitations need to be considered. First, the substantial reductions in problematic smartphone use were confirmed at a 3-week follow-up, but one cannot be sure whether there would be long-term maintenance of the improved behavior. Second, the primary outcome was a self-report assessment because objective data could not be obtained for this criterion. Third, further smartphone-related outcomes and mechanisms should be examined, such as smartphone-related impulsivity or habitual smartphone use [4]. Fourth, although the intervention condition included comprehensive theory-based content, this condition was not found to be superior in the final evaluation compared to the active control condition. Active control conditions benefit from the attention they received from the researchers or the software, and one can assume that volunteering participants come along with a high level of curiosity and motivation to succeed. However, a passive control condition was intentionally missing in this research design. It should be noted that the effects of a digital detox intervention, as opposed to a passive control condition, were examined by prior research [13]. Fifth, regarding sample characteristics, reactive recruitment procedures (eg, online postings and email lists) resulted in high participation rates of women and younger persons. Thus, associations of study variables with gender and age should be interpreted with caution. Moreover, the distribution of gender and age does not allow for inferences regarding the general population to be made. Further studies need to find representative samples for defined populations, for instance, by using proactive recruitment strategies. Finally, high attrition rates were observed, which is a general issue in online-based research [36]. Future studies could add just-in-time procedures matched to smartphone users’ needs to maintain user engagement [38].

### Conclusions

An innovative, theory-based intervention app was successfully evaluated as being capable of changing problematic smartphone use. The app was found to be useful for lowering problematic smartphone use and daily time spent with the device. However, observed reductions in both outcomes were not superior to those in the active control condition (ie, digital detox treatment). As an intervention mechanism, the intervention condition developed increased self-efficacy toward goal-directed smartphone use, which was linked to a reduction in problematic smartphone use. Further, planning of smartphone use at the end of the intervention phase was connected with a lower frequency of daily smartphone unlocks, which, in turn, was related to less problematic smartphone use. Further research could build on theories of behavior change and identify more psychological intervention content to improve these types of intervention apps. The app in its current form appears suitable for practical use as an alternative to common digital detox apps.

## Appendix

Multimedia Appendix 1 Supporting information on the study design and results.

Multimedia Appendix 2 Exemplary study material from the intervention app.

Multimedia Appendix 3 Audio-based exercise of Day 2.

Multimedia Appendix 4 CONSORT-eHEALTH checklist (V 1.6.1).

## Abbreviations

BCT: behavior change technique
CFI: comparative fit index
CIbc: bias-corrected bootstrap CI
CONSORT: Consolidated Standards of Reporting Trials
D: Day
HAPA: health action process approach
ICC: intraclass correlation
RCT: randomized controlled trial
RMSEA: root mean square error of approximation
SRMR: standardized root mean square residual
