# Supplementary material for: A Mobile Intervention for Self-Efficacious and Goal-Directed Smartphone Use in the General Population: Randomized Controlled Trial
Source: JMIR Mhealth Uhealth. 2021 Nov 23;9(11):e26397. doi: 10.2196/26397 (PMC8663477; doi:10.2196/26397)
Supplement: Multimedia Appendix 1 [file mhealth_v9i11e26397_app1.docx]

**Multimedia Appendix 1**

Table S1. Demographic information about the total sample and per condition

| Demographic information | Total sample  (*N* = 232) | Intervention condition (*n* = 114) | Active control condition (*n* = 118) |
| --- | --- | --- | --- |
| Gender, *n (%)* |  |  |  |
| Female | 205 (88.4) | 101 (88.6) | 104 (88.1) |
| Male | 23 (9.9) | 11 (9.6) | 12 (10.2) |
| Other | 1 (0.4) | 1 (0.9) | 0 (0.0) |
| Missing value | 3 (1.3) | 1 (0.9) | 2 (1.6) |
| Age | 29.62 (8.09) | 29.62 (8.09) | 30.08 (8.17) |
| Nationality, *n (%)* |  |  |  |
| German | 178 (76.7) | 93 (81.6) | 85 (72.0) |
| Swiss | 14 (6.0) | 7 (6.1) | 7 (5.9) |
| Austrian | 6 (2.6) | 2 (1.8) | 4 (3.4) |
| Russian | 4 (1.7) | 1 (0.9) | 3 (2.5) |
| British | 3 (1.3) | 2 (1.8) | 1 (0.8) |
| Italian | 3 (1.3) | 0 (0.0) | 3 (2.5) |
| Luxembourgish | 3 (1.3) | 2 (1.8) | 1 (0.8) |
| South Korean | 2 (0.9) | 1 (0.9) | 1 (0.8) |
| US-American | 2 (0.9) | 1 (0.9) | 1 (0.8) |
| Other Nationalities | 12 (5.2) | 3 (2.6) | 9 (7.6) |
| Missing value | 5 (2.2) | 2 (1.6) | 3 (2.5) |
| Highest education, *n (%)* |  |  |  |
| Doctorate Degree | 4 (1.7) | 2 (1.8) | 2 (1.7) |
| Masters degree | 54 (23.3) | 23 (20.2) | 31 (26.3) |
| Bachelor degree | 51 (22.0) | 24 (21.1) | 27 (22.7) |
| High school graduate | 98 (42.2) | 54 (47.4) | 44 (37.3) |
| Other degrees | 22 (9.5) | 10 (8.8) | 12 (10.2) |
| Missing value | 3 (1.3) | 1 (0.9) | 2 (1.6) |
| Employment, *n (%)* |  |  |  |
| Student | 105 (45.3) | 54 (47.4) | 51 (43.2) |
| Salaried | 90 (38.8) | 44 (38.6) | 46 (39.0) |
| Self-Employed | 22 (9.5) | 10 (8.8) | 12 (10.2) |
| Other | 12 (5.2) | 5 (4.4) | 7 (5.9) |
| Missing value | 3 (1.3) | 1 (0.9) | 2 (1.6) |

Note. (%): Percentage of respective samples.

Table S2. Descriptive statistics of main variables for both experimental conditions

| Study variables | Measurement occasion | Active Control Condition (*n* = 118) | | Intervention Condition (*n* = 114) | |
| --- | --- | --- | --- | --- | --- |
|  |  | *n* | *M (SD)* | *n* | *M (SD)* |
| Number of daily sessions (1-20) | D1-D20 | 118 | 11.34 (7.87) | 114 | 12.84 (7.41) |
| Perceived Impact (1-6) | D21 | 51 | 3.76^a^ (1.21) | 52 | 4.88^a^ (0.80) |
| Self-efficacy towards goal-directed smartphone use (1-6) | D1 | 113 | 4.53 (0.94) | 112 | 4.57 (1.05) |
|  | D20 | 41 | 4.41 (1.13) | 48 | 4.77 (0.88) |
| Planning of goal-directed smartphone use (1-6) | D1 | 115 | 2.34 (1.17) | 113 | 2.28 (1.23) |
|  | D16 | 44 | 3.31 (1.45) | 54 | 3.65 (1.42) |
|  | D20 | 42 | 3.26^b^ (1.47) | 48 | 3.93^b^ (1.33) |
| Smartphone use (minutes per day) | D1 | 103 | 218.07 (83.79) | 101 | 227.52 (90.57) |
|  | D21 | 45 | 180.62 (71.00) | 47 | 191.45 (85.01) |
|  | D42 | 40 | 175.33 (73.69) | 42 | 195.90 (92.70) |
| Smartphone unlocks per day | D1 | 104 | 78.18 (41.80) | 102 | 83.77 (44.85) |
|  | D21 | 39 | 80.05 (38.18) | 46 | 75.89 (29.79) |
|  | D42 | 41 | 79.07 (41.66) | 43 | 71.23 (35.42) |
| Problematic smartphone use (1-6) | D1 | 117 | 3.60 (0.80) | 113 | 3.65 (0.74) |
|  | D21 | 54 | 2.30 (0.85) | 54 | 2.12 (0.73) |
|  | D42 | 44 | 2.14 (0.65) | 44 | 2.06 (0.62) |

Note. ‘D’ refers to day of the study. Significant differences: a: Perceived impact higher in intervention condition [*F*(1, 101) = 30.53, *P* < .001, *η²* = 0.23]; b: Planning higher in intervention condition [*F*(1, 88) = 5.03, *P* = .027, *η²* = 0.05].

## Enrollment

Registration (*n* = 420)

Excluded (*n* = 188)

♦ did not download app (*n* = 151) ♦ did not complete baseline (*n* = 37)

Randomization (*N* = 232)

## Allocation (D1)

Allocated to active control condition (*n* = 118)

♦ Received allocated intervention (*n* = 118 )

Allocated to intervention condition (*n* =114)

♦ Received allocated intervention (*n* = 114 )

## Follow-Up (D42)

Attrition prior to D42: *n* = 11

Analysed (*n* = 44)

Attrition prior to D21: *n* = 59

Analysed (*n* = 55)

## Post Intervention (D21)

Attrition prior to D21: *n* = 63

Analysed (*n* = 55)

Attrition prior to D42: *n* = 11

Analysed (*n* = 44)

Figure S1. Flow diagram outlining participant allocation into the intervention condition or the active control condition.

Note. ‘D’ refers to day of the study.


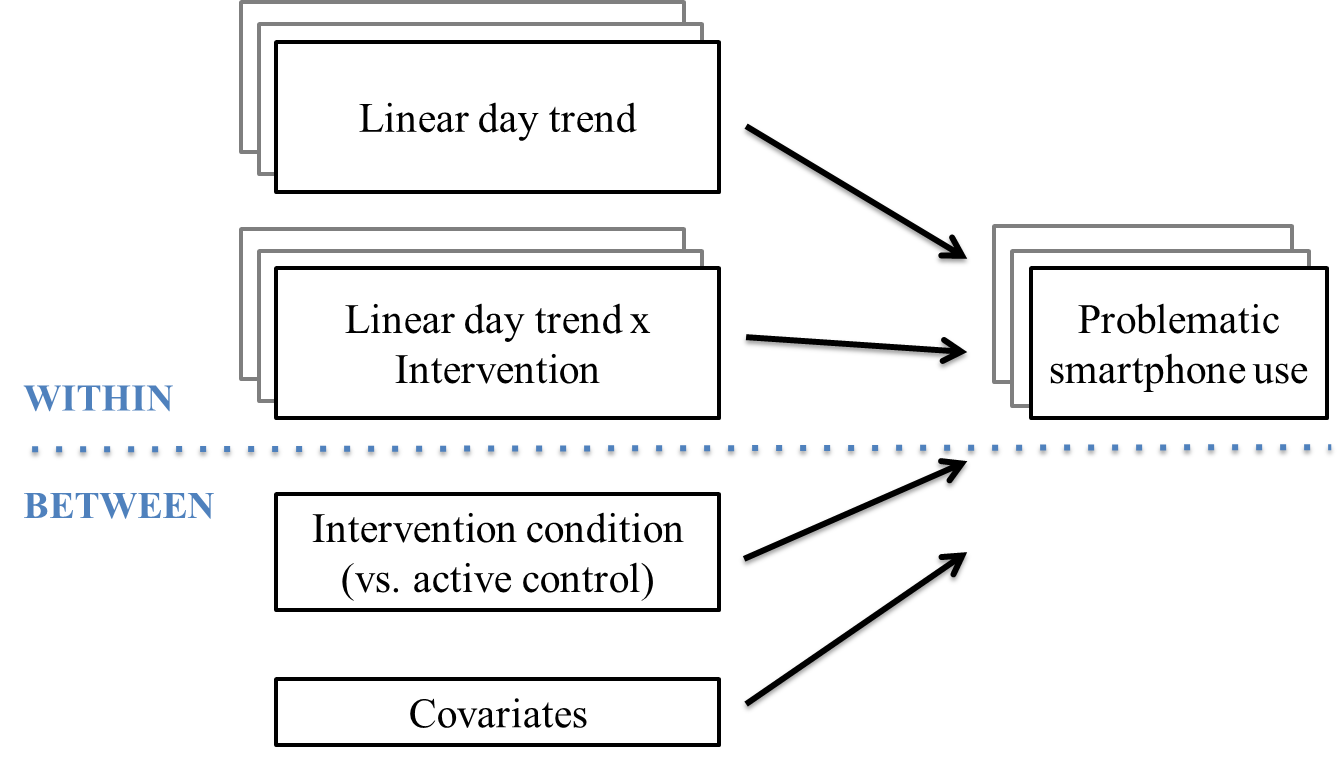


Figure S2. Conceptual two-level model with three time points (D1, D21, and D42; within level) nested in participants (between level) predicting within-level problematic smartphone use.

| 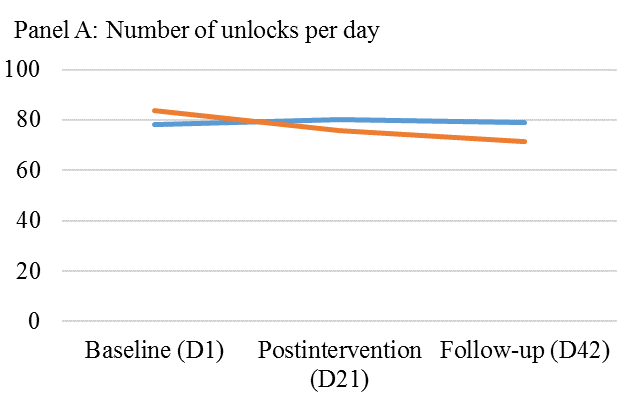 | 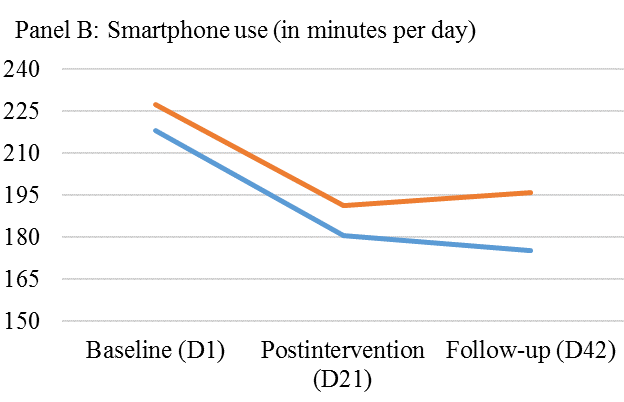 |
| --- | --- |
| 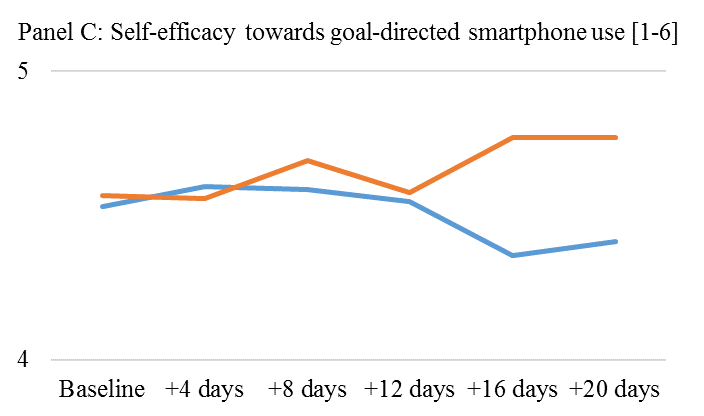 | 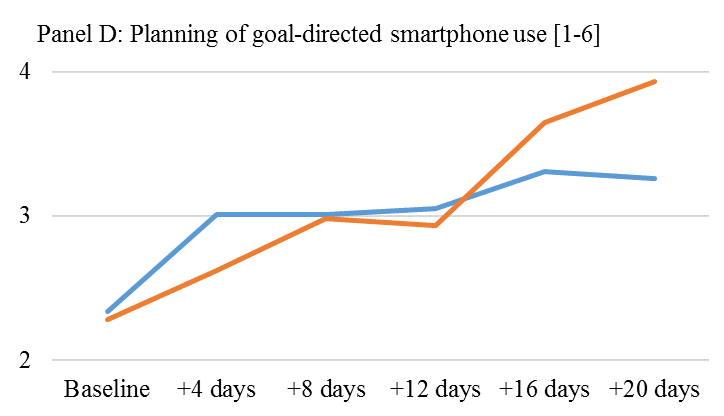 |

Figure S3. Mean levels over time of additional variables in the active control (blue) and intervention (orange) conditions.

Note. ‘D’ refers to ‘Day’ of the study. Only parts of the response scale were displayed in Panels B, C, and D.
