# Supplementary material for: A Mobile Intervention for Self-Efficacious and Goal-Directed Smartphone Use in the General Population: Randomized Controlled Trial
Source: JMIR Mhealth Uhealth. 2021 Nov 23;9(11):e26397. doi: 10.2196/26397 (PMC8663477; doi:10.2196/26397)
Supplement: Multimedia Appendix 2 [file mhealth_v9i11e26397_app2.pdf]

Multimedia Appendix 2
Daily exercises of the intervention condition, exemplary for:
Day 2 (module 1)
Day 7 (module 2)
Day 10 (module 3)
Day 13 (module 4)
Day 20 (module 5)

Take on a good posture

●●●○○ GS 9:41 AM 58 %

not less
but better

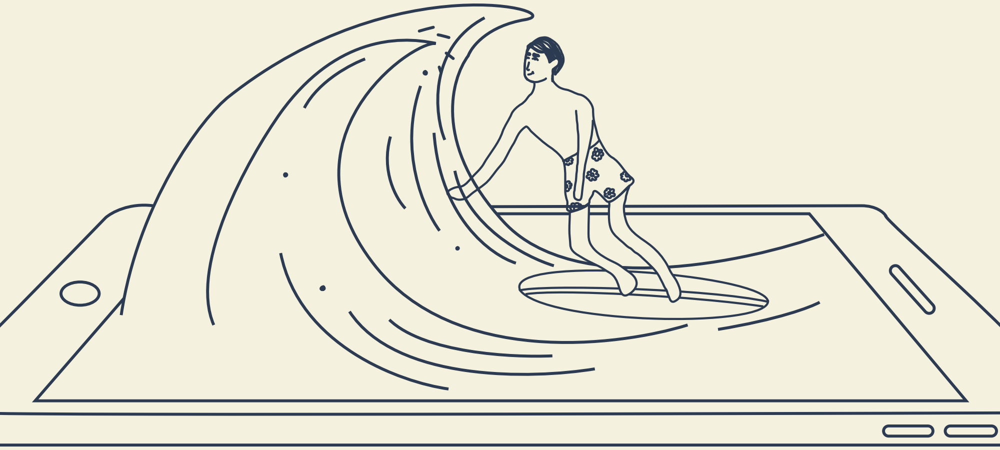

ACCESS YOUR ACCOUNT

●●●○○ GS 9:41 AM 58 %

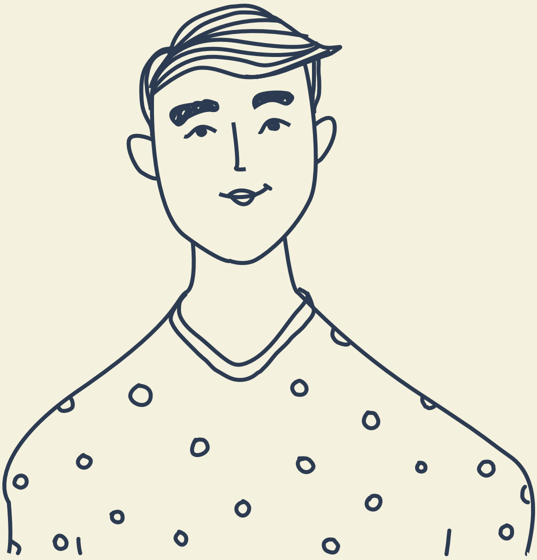

Hi, I’m Leo.

And I’m going to be your companion on this journey. How do you like to be called?

Your Nickname

NEXT

●●●○○ GS 9:41 AM 58 %

Hi, I’m Leo.

And I’m going to be your companion on this journey. How do you like to be called?

Andre

NEXT

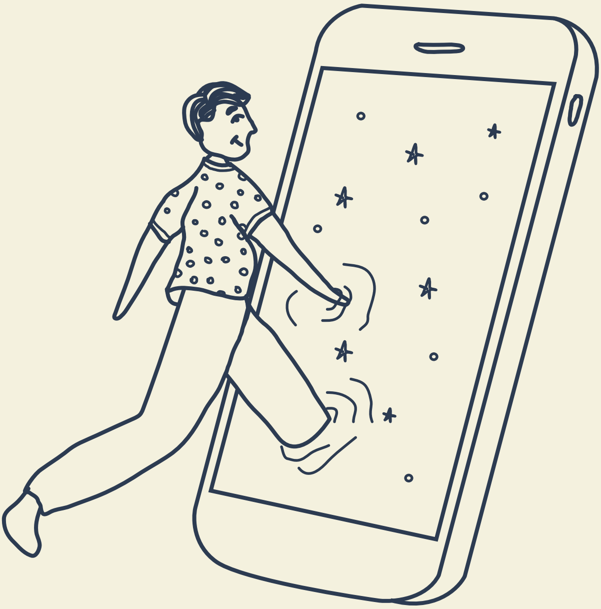

You can probably relate to my situation, {username}: I love my smartphone. It opens up a whole new world right at my fingertips, just like a big ocean full of great possibilities.

●●●○○ GS 9:41 AM 58 %

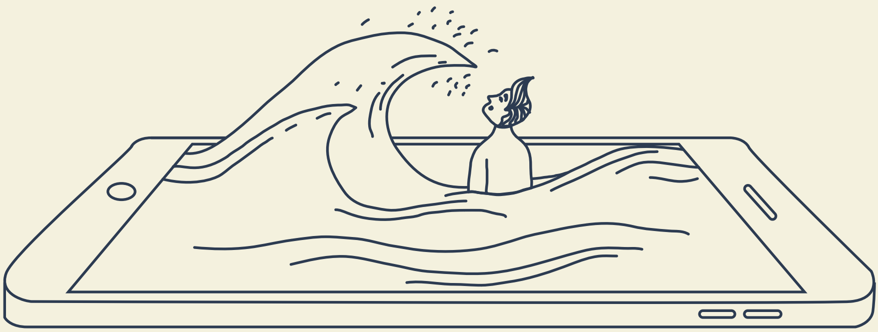

But the ocean is not calm. There are lots of stormy waves of distraction that pull you underwater. Sometimes, I feel like almost drowning. And I'm not alone: many people feel that way.

●●●○○ GS 9:41 AM 58 %

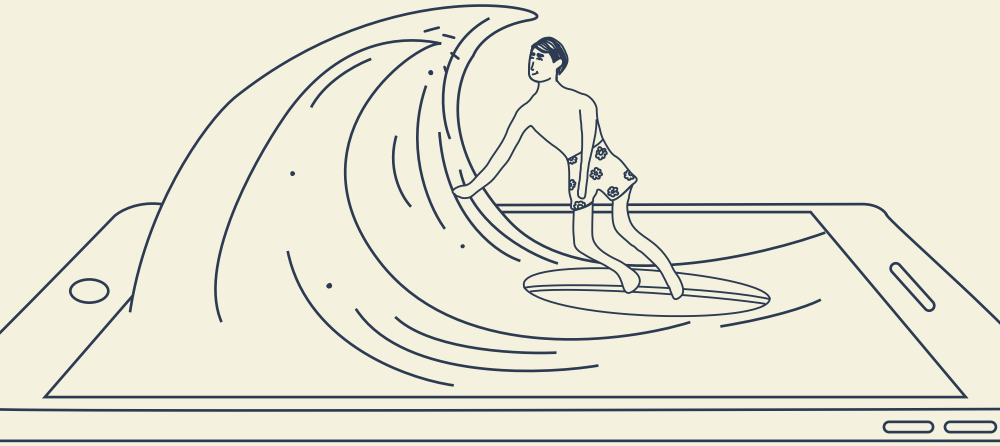

This program is about mastering the waves.

In 21 days, you will learn how to develop a healthy relationship to your smartphone. One exercise per day. No more feelings of drowning.

NEXT

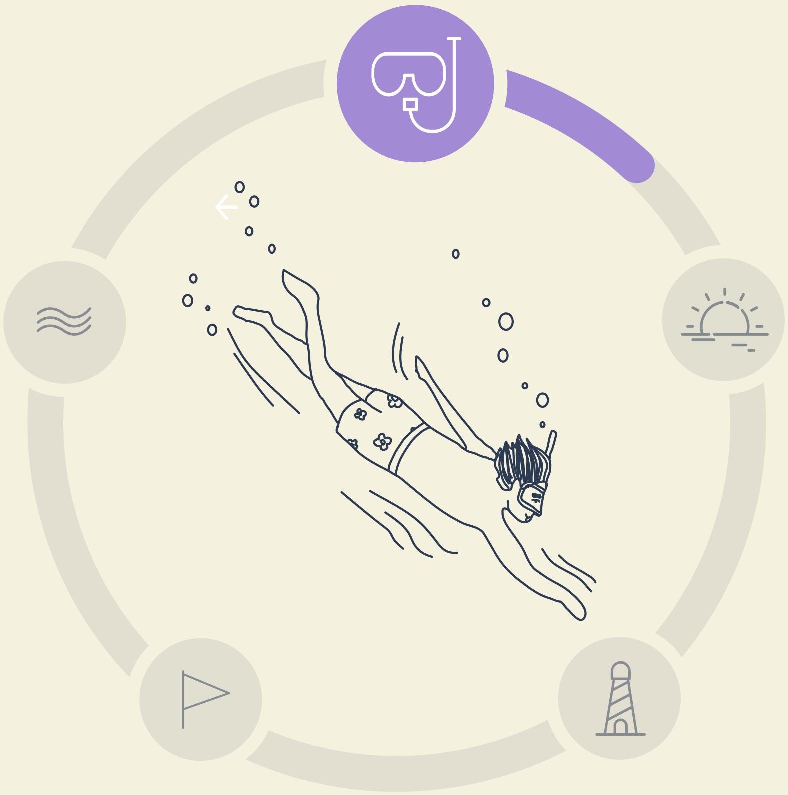

Hey, Andre!

Continue with your next session.

Day 2 - Notice Your Impulses

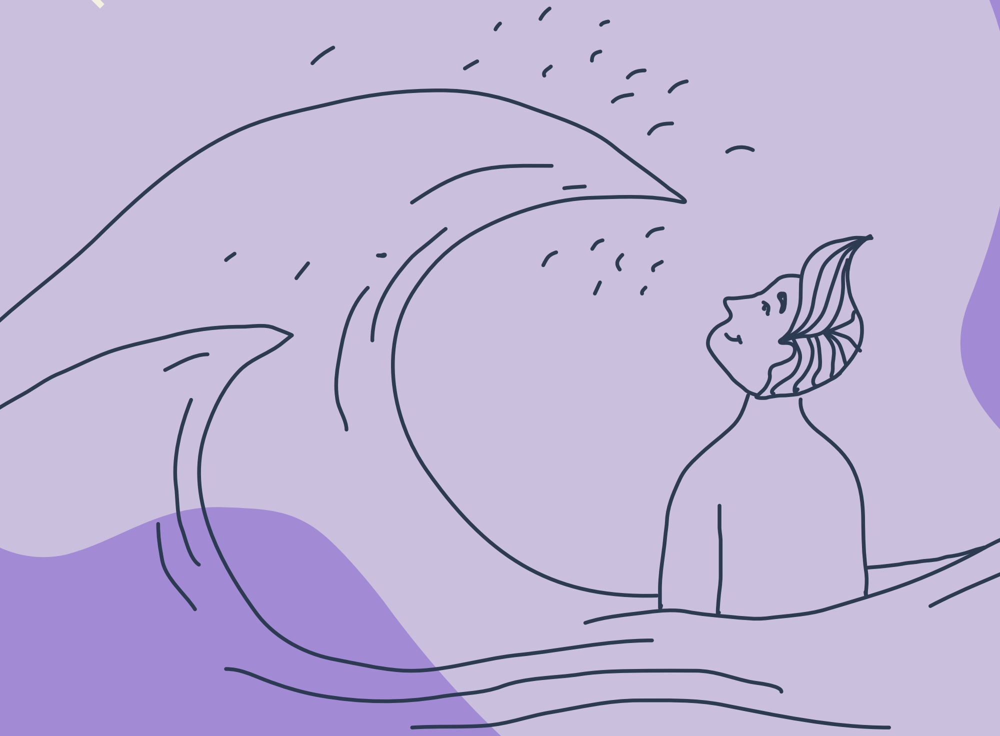

Day 2

Notice Your Impulses

4 MIN

Today, you will discover the unconscious force that pushes you to your smartphone.

START

Notice Your Impulses

Notice Your Impulses

15

15

02:50

10:00

Aha!

Impulses are one of the most important drivers of smartphone use. Therefore, they provide a great starting point for change.

Impulses are like waves: they come over us and without realizing, they sweep us to wherever they want.

Aha!

Learning to detect the impulse and noticing it, for example by mentally saying “Now, I have an impulse to use the smartphone” is your first step towards conscious smartphone use.

The Challenge

Whenever you catch yourself grabbing your smartphone, stop for a moment and try to see if you can identify the impulse. Notice it with curiosity and without judging it. Practice this until tomorrow.

Try to let the impulse come over you, like a wave, without reacting to it immediately. And then decide consciously if you want to follow your impulse right now - or not.

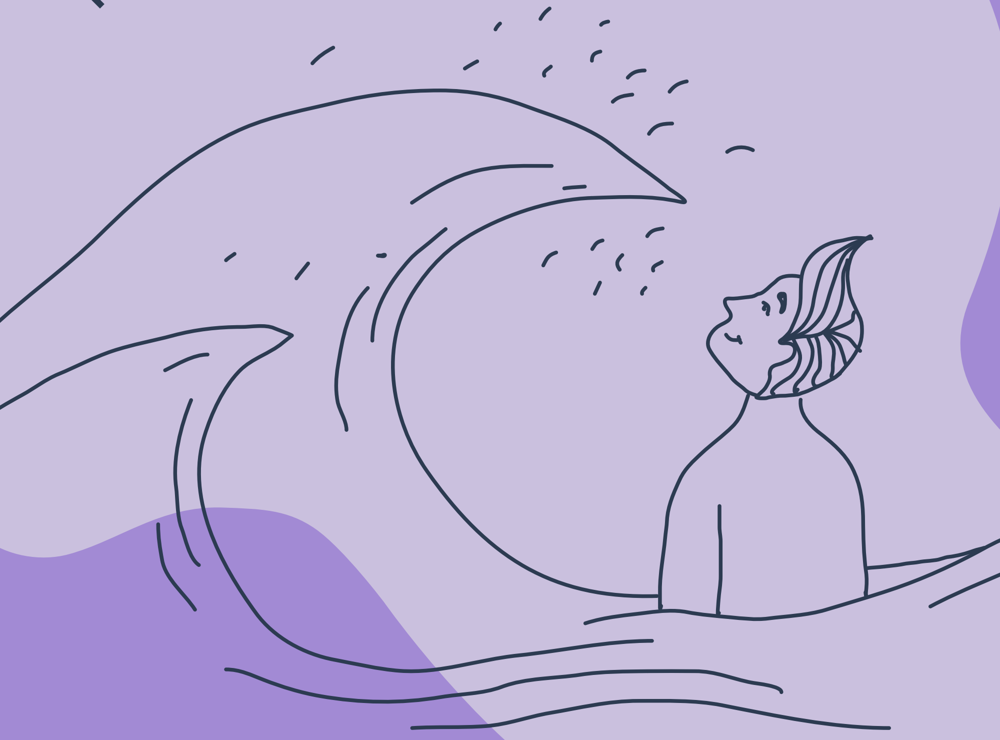

Congratulations!

You just completed the first step towards a more conscious smartphone use.

DONE

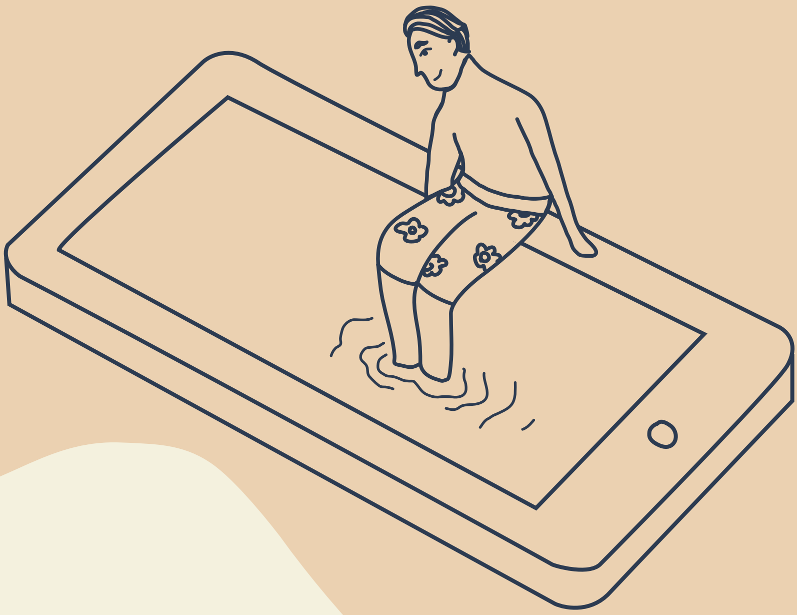

Reflect

Studying the waves
You might know the feeling of being hooked to your smartphone and losing yourself in it. It's like getting hit by a big wave in the ocean that pulls you underwater.

Leo is standing at the beach. With a bit of distance, he tries to understand the dynamics of the impulse waves in the digital ocean.

This module is about understanding the drivers of your unconscious smartphone use. Explore why it's so hard to resist the pull and discover how to regain control over your [illegible]

TAP ANYWHERE TO CONTINUE

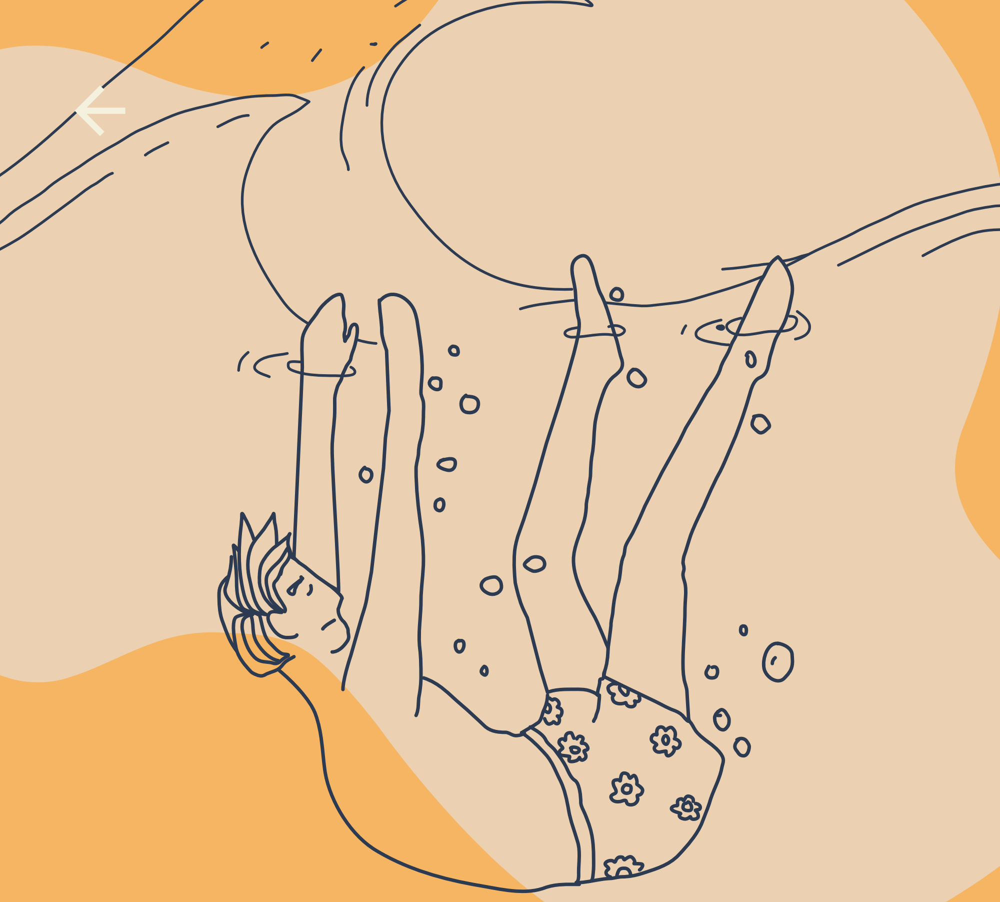

Day 7

Getting Lost

4 MIN

In this exercise, you will discover why you sometimes get lost while using your smartphone.

START

Which apps are your personal danger zones that pull you in?

In order to find out, check your most used apps in your settings.

For iOs: Settings > Screen Time > {username}s iPhone > Last 7 day > scroll down to MOST USED

For Android: Settings > Digital Well-Being > Dashboard > scroll down

If you do not use a screen time tracker, just reflect on this for a moment.

Which three apps do you give a red light?

The ones that you catch yourself spending more time on than you intend to.

Twitter

Facebook

Instagram

Whatsapp

Pinterest

Safari

Netflix

Youtube

Google Maps

Spotify

Soundcloud

Audible

Alarm Clock

Calculator

Headspace

Calm

Write a new one +

CONTINUE

Which three apps do you give a red light?

The ones that you catch yourself spending more time on than you intend to.

Twitter
Facebook
Instagram
Whatsapp
Pinterest
Safari
Netflix
Youtube
Google Maps
Spotify
Soundcloud
Audible
Alarm Clock
Calculator
Headspace
Calm

Write a new one +

CONTINUE

Which three apps do you give a green light?

They are the ones you rarely or never spend more time on than intended.

Twitter
Facebook
Instagram
Whatsapp
Pinterest
Safari
Netflix
Youtube
Google Maps
Spotify
Soundcloud
Audible
Alarm Clock
Calculator
Headspace
Calm

Write a new one +

CONTINUE

Which three apps do you give a green light?

They are the ones you rarely or never spend more time on than intended.

Twitter **Facebook** Instagram

Whatsapp Pinterest Safari

Netflix Youtube **Google Maps**

Spotify **Soundcloud** Audible

Alarm Clock Calculator Headspace

Calm

Write a new one +

CONTINUE

Okay. But, how do ***Facebook, Google Maps, and Soundcloud*** actually pull you in?

Don’t worry, it’s not you, it’s them.

Aha!

The apps that tend to draw us in the most share the same design features to keep our attention hooked. What these apps have in common is that they don't have any stopping cues, that act as anchors in an infinite sea of content.

Aha!

What is a Stopping Cue?

A stopping cue is a signal showing us that it might be time to move on. When you read a book and reach the last page of the chapter, then this is the signal that you can easily stop here. You have reached an end-point. Or when you watch a movie in the theater, you know that it is time to leave when the movie is finished.

Aha!

In the digital world, stopping cues are blurrier than ever. Knowing that it is time to stop is not as easy anymore as reaching the end of a chapter and closing a book. In many apps, you can simply scroll, swipe or be on autoplay forever. They are bottomless: there will simply not be an end.

The Challenge

When you continue using your smartphone today, think of the traffic light and the apps you use. How does their design add to the experience of losing yourself in the app?

Become aware of that.

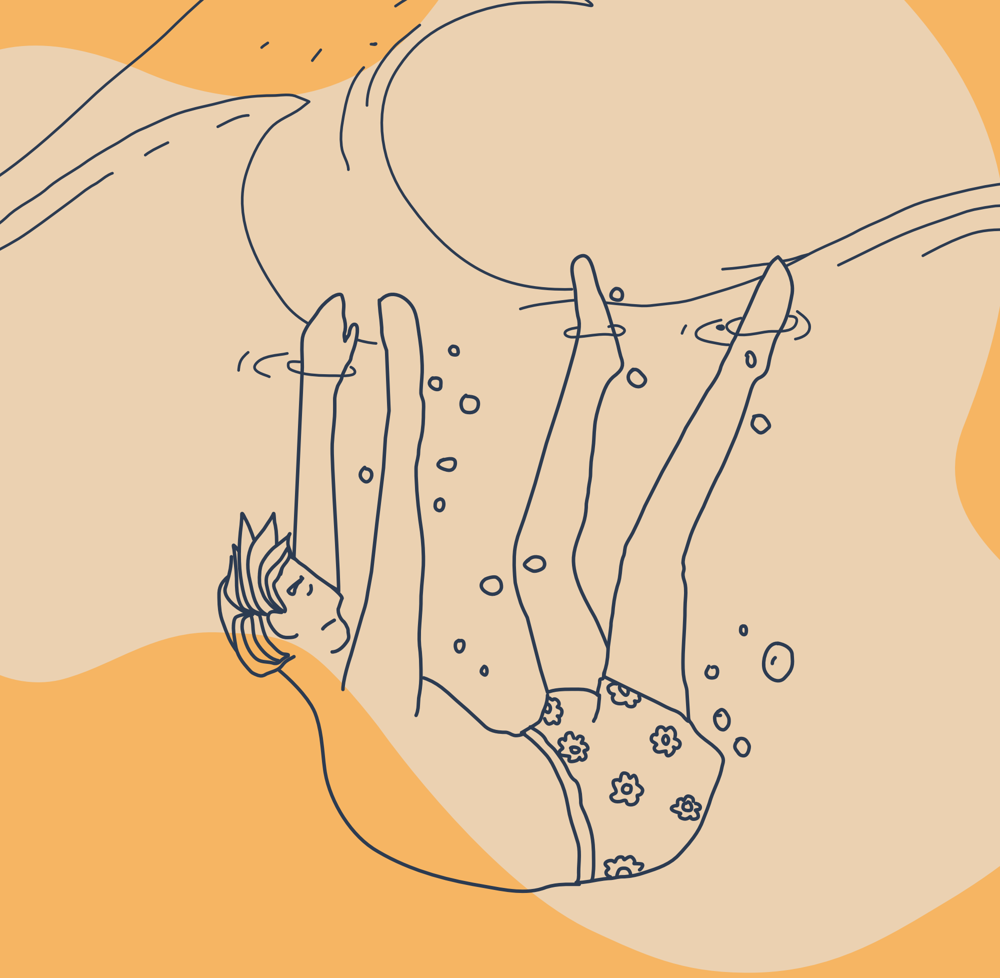

Congratulations!

You now know how bottomless apps are designed to pull you in and make you get lost in them.

DONE

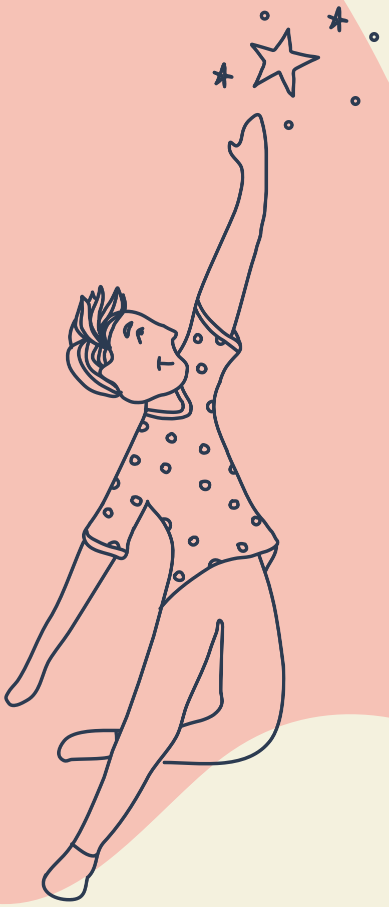

Vision

Finding meaning
No matter how good you can surf: if you're unsure about the direction you're heading, you will eventually go down. It's similar to your smartphone use: if you know what's important to yourself, you're more likely to handle mindless scrolling on the screen.

Leo learned why some waves soak him underwater. Suddenly, he sees a bright light on the horizon and turns his head towards the lights. It's a big star! Leo is fascinated by the power of the star and wants to find out more about it. So he does the obvious: he reaches for the star.

This module is about exploring your values. Based on them, you will define a new way to use your [illegible]

TAP ANYWHERE TO CONTINUE

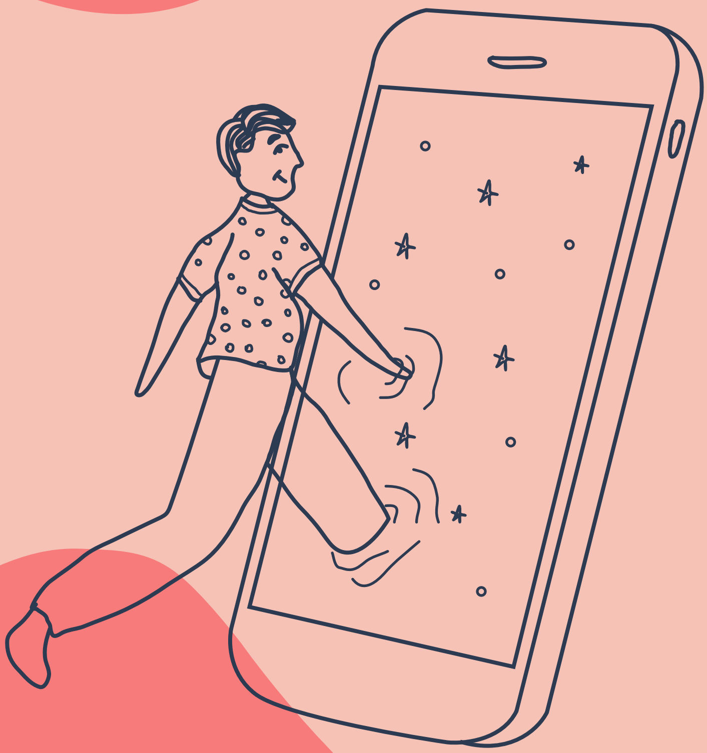

Day 10

Meaningful Smartphone Activities

5 MIN

Today, you are going to turn your smartphone into a tool to bring your values to life.

START

How can you realize what is important to you and bring your values to life?

Your three values are:

{Value1_9}

{Value2_9}

{Value3_9}

TAP ANYWHERE TO CONTINUE

We will have a look at Leo’s values first.

What could activities look like that bring his values to life?

Learning → Reading a science magazine

Relatedness → Having coffee with his best friends

Gratefulness → Gifting flowers to someone

What could activities on his smartphone look like that put his values into practice?

Learning → Listening to a TED talk on his smartphone

Relatedness → Sending his best friend a thoughtful message

Gratefulness →starting a gratefulness journal in the notes

Bringing values to life

What could an activity look like that helps you bring *{Value1_9}* to life?

E.g. Learning → → Reading a science magazine

Write down your activity

CONTINUE

Q W E R T Y U I O P
A S D F G H J K L
Z X C V B N M
123 space return

Bringing values to life

What could an activity look like that helps you bring *{Value1_9}* to life?

E.g. Learning → → Reading a science magazine

Talk to people

CONTINUE

Q W E R T Y U I O P
A S D F G H J K L
Z X C V B N M
123 space return

Bringing values to life with your smartphone

What could an activity on your smartphone look like that helps you bring *{Value1_9}* to life?

E.g. Learning → Listening to a TED talk on his smartphone

Write down your activity

CONTINUE

Q W E R T Y U I O P
A S D F G H J K L
Z X C V B N M
123 space return

Bringing values to life with your smartphone

What could an activity on your smartphone look like that helps you bring *{Value1_9}* to life?

E.g. Learning → Listening to a TED talk on his smartphone

Talk to people

CONTINUE

Q W E R T Y U I O P
A S D F G H J K L
Z X C V B N M
123 space return

Bringing values to life

What could an activity look like that helps you bring {Value2_9} to life?

E.g. Relatedness → Having coffee with his best friends

Write down your activity

CONTINUE

Q W E R T Y U I O P
A S D F G H J K L
Z X C V B N M
123 space return

Bringing values to life

What could an activity look like that helps you bring *{Value2_9}* to life?

E.g. Relatedness → Having coffee with his best friends

Talk to people

CONTINUE

Q W E R T Y U I O P
A S D F G H J K L
Z X C V B N M
123 space return

Bringing values to life with your smartphone

What could an activity on your smartphone look like that helps you bring *{Value2_9}* to life?

E.g.Relatedness → Sending his best friend a thoughtful message

Write down your activity

CONTINUE

Q W E R T Y U I O P

A S D F G H J K L

Z X C V B N M

123 space return

← ✕

Bringing values to life with your smartphone

What could an activity on your smartphone look like that helps you bring *{Value2_9}* to life?

E.g. Relatedness → Sending his best friend a thoughtful message

Listen to the daily TED talk

CONTINUE

Q W E R T Y U I O P
A S D F G H J K L
Z X C V B N M
123 space return

Bringing values to life

What could an activity look like that helps you bring *{Value3_9}* to life?

E.g.Gratefulness → Gifting flowers to someone

Write down your activity

CONTINUE

Q W E R T Y U I O P
A S D F G H J K L
Z X C V B N M
123 space return

The Challenge

Until tomorrow, do a test run of the meaningful smartphone activity that you defined today!

Your meaningful activities are:

Activity in the smarthone 1
for *value 1*.

Activity in the smarthone 2
for *value 2*.

Activity in the smarthone 3
for *value 3*

Aha!

Research shows: What we do on our screens is more important than how much time we spend on them!

TAP ANYWHERE TO CONTINUE

Aha!

By transforming values into action, you can boost the meaningfulness of your screen time and make the smartphone a tool for living closer to your values in everyday life.

Later in the program, we will revisit this activity and see how you can work towards establishing them as new habits.

The Challenge

Until tomorrow, do a test run of the meaningful smartphone activity that you defined today!

Your meaningful activities are:

{MeanSmAct1_10}

{MeanSmAct2_10}

{MeanSmAct3_10}

←

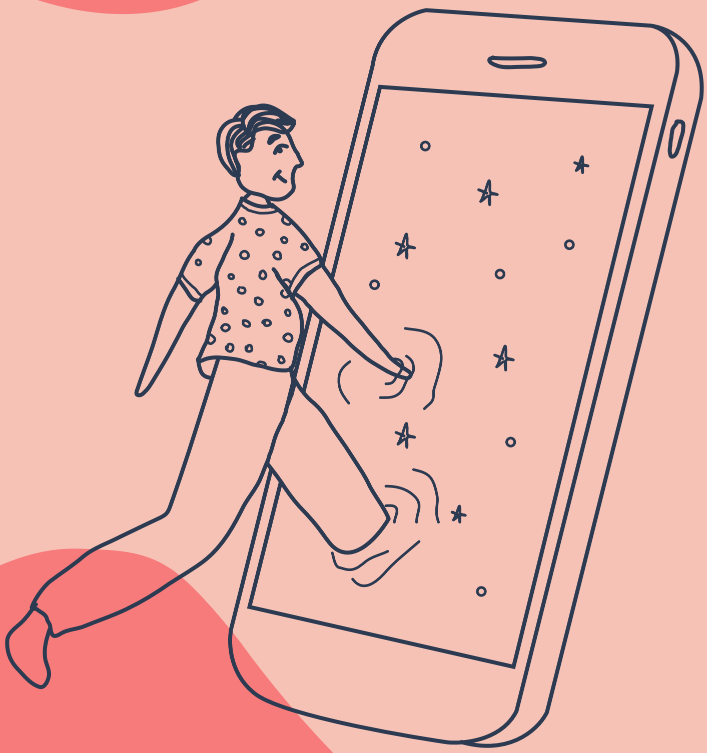

Congratulations!

Today, you discovered how your smartphone can be a tool to put your values into practice!

DONE

●●●○○ GS 9:41 AM 58 %

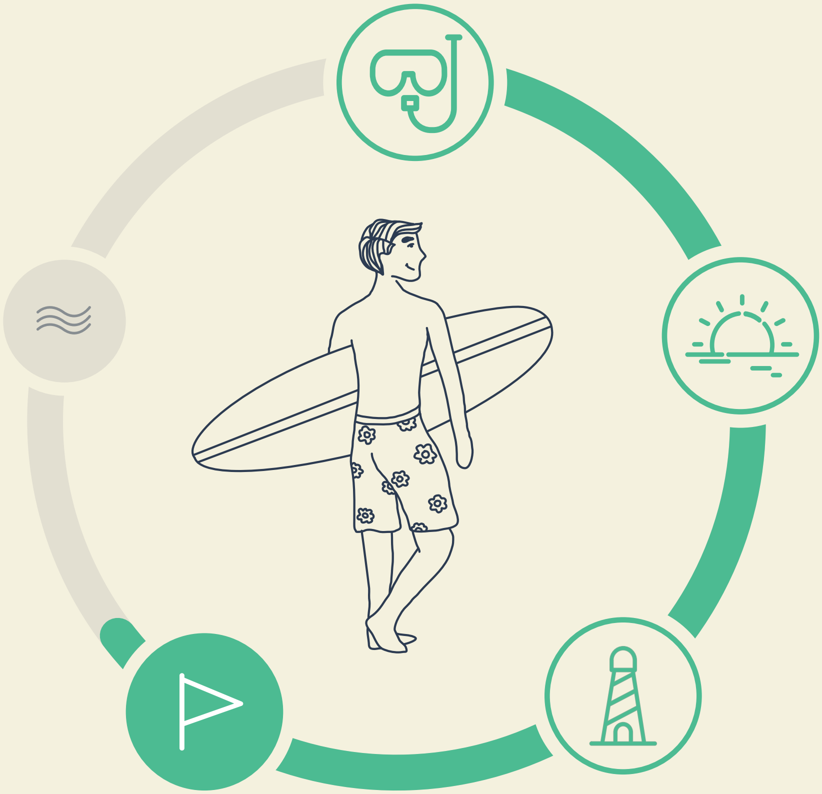

Hey, Andre!

Continue with your next session.

Day 13 - Meaningful Smartphone Habits ▶

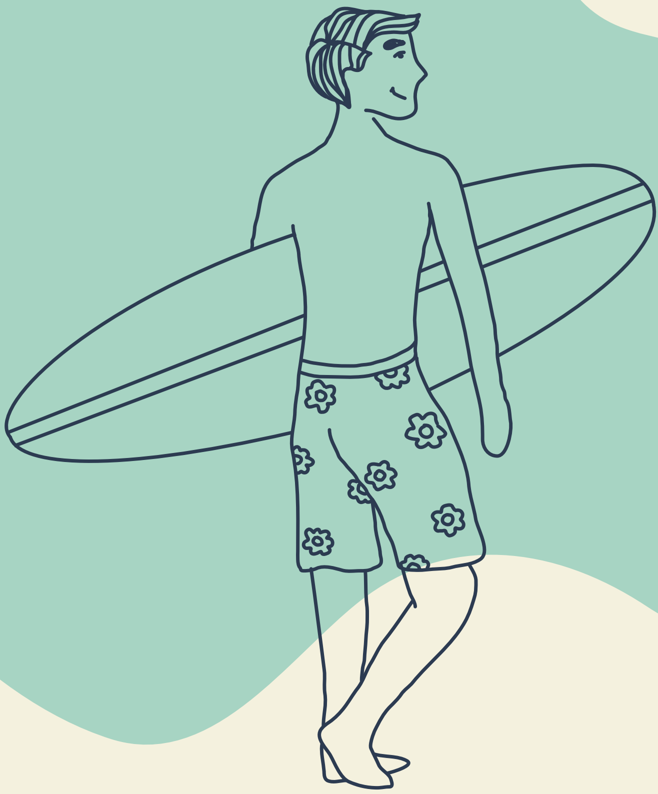

Plan

Preparing for the waves
Turns out that values are the secret of success to handle the waves! Having learned this on the beach, Leo is back on his way to the ocean. It’s time to prepare for the big challenge: mastering and surfing the waves.

This module is about introducing meaningful smartphone habits. The goal is to learn new ways to react to impulses. Prepare for the rough part of the digital ocean and discover how to surf the impulse waves.

TAP ANYWHERE TO CONTINUE

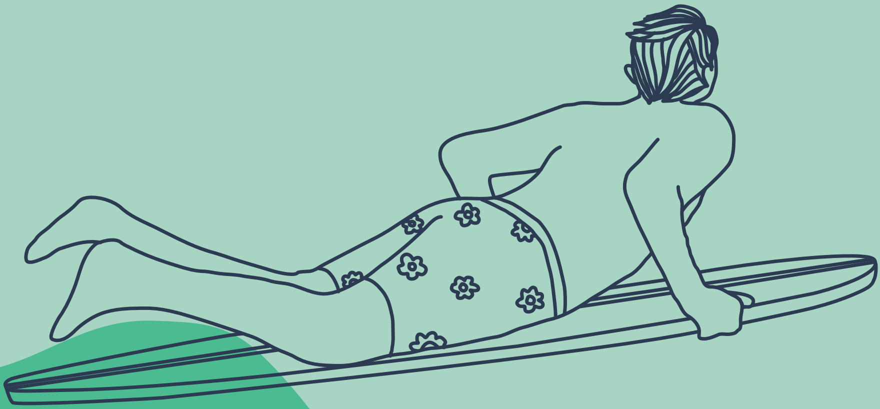

Day 13

Meaningful Smartphone Habits

5 MIN

In this exercise, you will discover how to make good plans.

START

You will build a powerful new smartphone habit using the following recipe.

The beauty of it: during this journey, you have already defined all elements you need for it. Now it’s just about putting them together!

TAP ANYWHERE TO CONTINUE

Step 1: Choose three triggers

For which situations - or feelings - do you want to define a new response?

View example

If I am...

{EmTrigger1_D6}

{EmTrigger2_D6}

{EmTrigger3_D6}

{SitTrigger1_D6}

{SitTrigger2_D6}

{SitTrigger3_D6}

CONTINUE

Step 1: Choose three triggers

For which situations - or feelings - do you want to define a new response?
View example

If I am...

{EmTrigger1_D6}

{EmTrigger2_D6}

{EmTrigger3_D6}

{SitTrigger1_D6}

{SitTrigger2_D6}

{SitTrigger3_D6}

CONTINUE

E.g. Leo, for which situation - or feeling - do you want to define a new response?

If I am in the metro...

If I am lonely...

Step 2: Choose your new behavior

What would you like to do differently in this situation? View example

If I am ***{NewHabitTrigger1_D13}***, then I will...

{MeanSmAct1_D10}

{MeanSmAct2_D10}

{MeanSmAct3_D10}

CONTINUE

Step 2: Choose your new behavior

What would you like to do differently in this situation? View example.

If I am ***{NewHabitTrigger1_D13}***, then I will...

{MeanSmAct1_D10}

{MeanSmAct2_D10}

{MeanSmAct3_D10}

CONTINUE

E.g. ... then I will listen to a TED talk on my smartphone.

...then I will send my best friend a thoughful message.

Step 2: Choose your new behavior

What would you like to do differently in this situation? View example.

If I am ***{NewHabitTrigger2_D13}***, then I will...

{MeanSmAct1_D10}

{MeanSmAct2_D10}

{MeanSmAct3_D10}

CONTINUE

Step 2: Choose your new behavior

What would you like to do differently in this situation? View example.

If I am ***{NewHabitTrigger2_D13}***, then I will...

{MeanSmAct1_D10}

{MeanSmAct2_D10}

{MeanSmAct3_D10}

CONTINUE

Step 2: Choose your new behavior

What would you like to do differently in this situation? View example.

If I am ***{NewHabitTrigger3_D13}***, then I will...

{MeanSmAct1_D10}

{MeanSmAct2_D10}

{MeanSmAct3_D10}

CONTINUE

Step 2: Choose your new behavior

What would you like to do differently in this situation? View example.

If I am ***{NewHabitTrigger3_D13}***, then I will...

{MeanSmAct1_D10}

{MeanSmAct2_D10}

{MeanSmAct3_D10}

CONTINUE

Step 3: Commit to a new habit

These are your three freshly designed plans for meaningful smartphone habits.

Let’s take it step by step. Pick one of the plans you want to establish first.

If am *{NewHabitTrigger1_D13}*, then I will *{NewHabitActivity1_D13}*

If am {NewHabitTrigger2_D13}, then I will {NewHabitActivity2_D13}

If am {NewHabitTrigger3_D13}, then I will {NewHabitActivity3_D13}

CONTINUE

Step 3: Commit to a new habit

These are your three freshly designed plans for meaningful smartphone habits.

Let's take it step by step. Pick one of the plans you want to establish first.

If am *{NewHabitTrigger1_D13}*, then I will *{NewHabitActivity1_D13}*

If am {NewHabitTrigger2_D13}, then I will {NewHabitActivity2_D13}

If am {NewHabitTrigger3_D13}, then I will {NewHabitActivity3_D13}

CONTINUE

Aha!

About 90% of new year resolutions fail. Having a gap between knowing what you want to do and actually getting it done is very human.

TAP ANYWHERE TO CONTINUE

Aha!

No willpower? No problem.

Fortunately, there is a powerful tool to help you achieve any goal and that looks like this:
If X happens, then I will do Y.

Why are these plans so effective?

Because they are **written in the language of your brain** – the language of contingencies.

We are particularly good at encoding and remembering information in “If X, then Y” terms to guide our behavior, often without our awareness.

The Challenge

Let’s walk the walk. Do a first run until tomorrow.

{NewHabitCommit_D13}

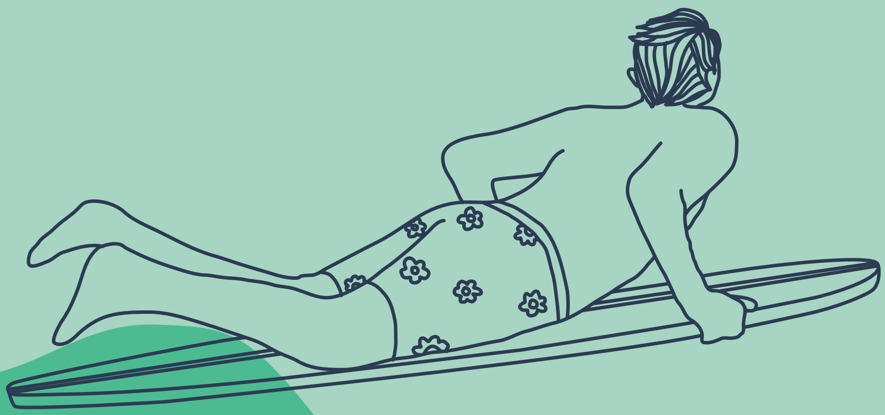

Congratulations!

Now you know the secret If-then recipe for new habits.

DONE

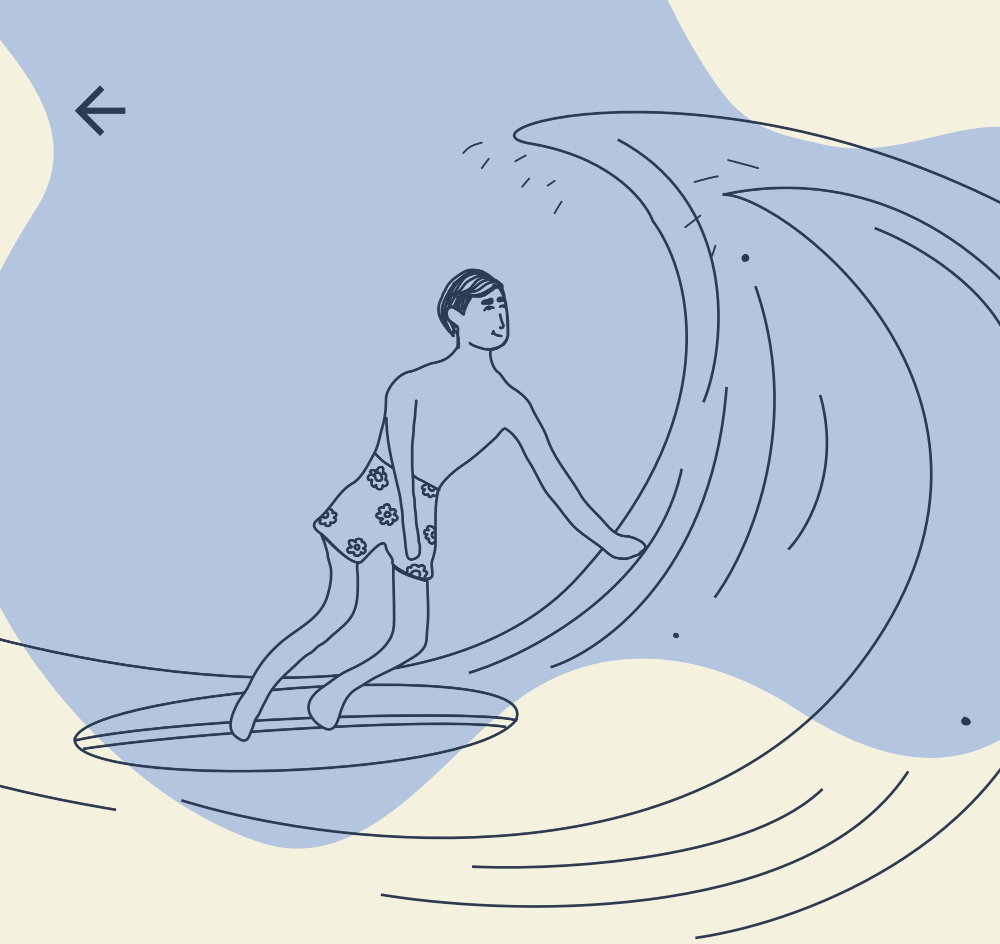

Support

Mastering the waves
It’s time to surf! In the last 16 days, Leo learned how to handle the stormy waves of distraction in the digital ocean. Now it’s about refining his surfing gear to make sure it supports him in mastering and surfing the waves.

This module is about redesigning your smartphone and environment to support your new smartphone practice. Create the perfect surf environment!

TAP ANYWHERE TO CONTINUE

●●●○○ GS 9:41 AM 58 %

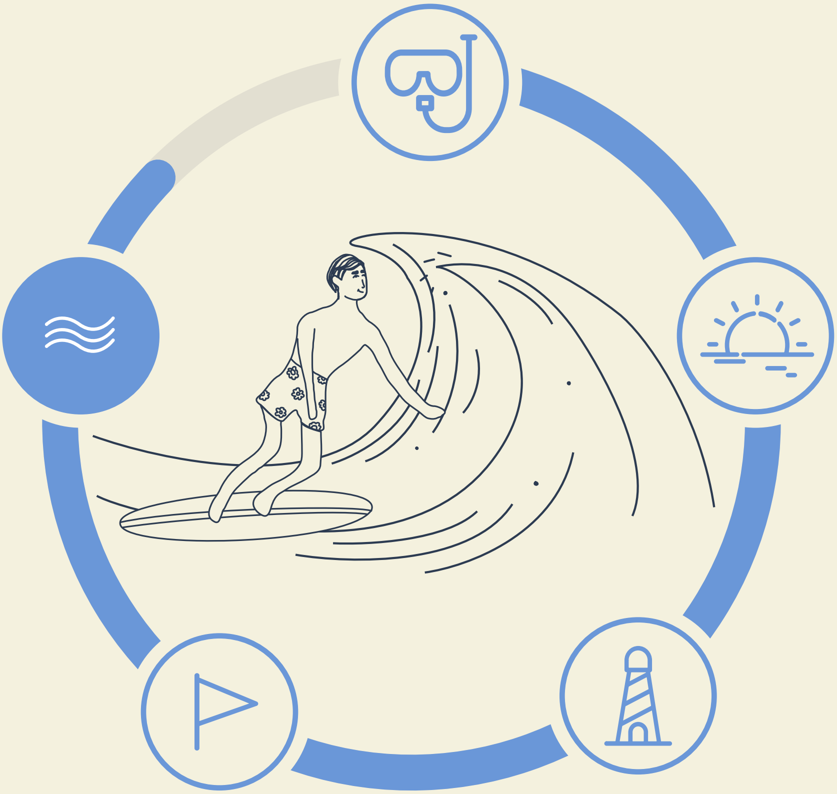

Hey, Andre!

Continue with your next session.

Day 20 - Daddeln is Ok ▶

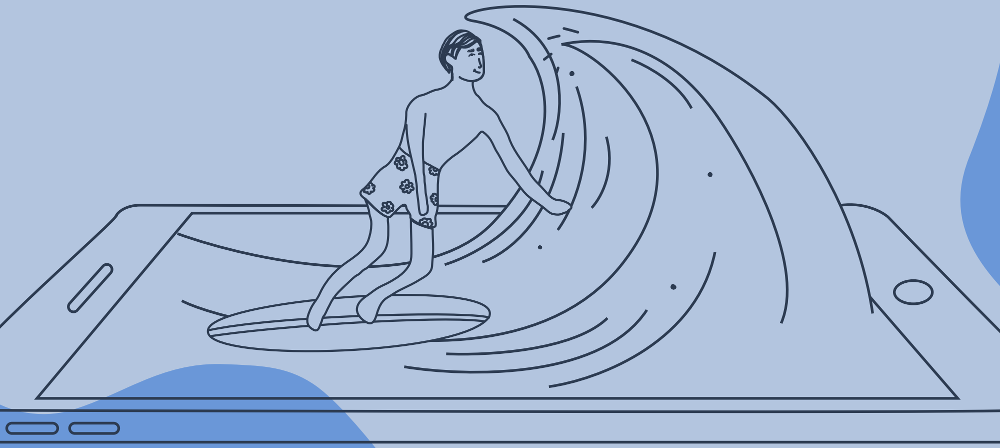

Day 20

Daddeln is Okay

7 MIN

Today, you’re ready to lose yourself again! Also, you’ll do your fifth reflection.

START

Wow!

You’re almost done with the 21-day training and you’ve learned a lot about yourself and how to consciously use your smartphone.

TAP ANYWHERE TO CONTINUE

Now, do your favourite mindless activity on your smartphone for five minutes.

This might be ***{AppsRed1_D7}***, ***{AppsRed2_D7}***, or ***{AppsRed3_D7}***.

Enjoy it to the fullest!

Start the timer in the next screen.

5:00

We'll let you know when it's time to come back.

START AND ENJOY

4:59

We'll let you know when it's time to come back.

0:00

Time is up. Continue the exercise.

CONTINUE

Welcome back! Wait a second? Wasn’t the aim of this whole course to do less of exactly this?

Doing mindless activities on your smartphone, following your impulses and losing yourself into something can be wonderful - under one condition: you know how to stop.

TAP ANYWHERE TO CONTINUE

Aha!

We call this activity **Daddeln.**

Most of us are doing Daddeln almost every day like mindlessly scrolling through Instagram or binge-watching YouTube after a long day of work.

At the beginning, this feels incredibly good to exhausted brains. But often, there comes a turning point, where the activity starts making us feel bad. We feel like we’ve spent more time on the device than we intended to.

Aha!

Luckily, there's a solution for fun Daddeln time: stopping cues!

A self-set stopping cue marks the end of a time frame in which we allow ourselves to follow our impulses.

That way, you can lose yourself without losing control.

Aha!

There are different stopping cues that you can use:

1. Time: I will do Daddeln for ten minutes and set an alarm.

2. Numbers: I will do Daddeln and watch two videos.

3. Situations: I will do Daddeln while commuting from A to B.

Aha!

Once you've made the decision to do Daddeln, you're free to enjoy the activity to the fullest.

Daddeln is supposed to make you feel good, not bad. No judgement at all, do what makes you feel good.

The Challenge

Do one more Daddeln session until tomorrow. Lose yourself and have fun!
